# Supplementary material for: Public attitudes value interpretability but prioritize accuracy in Artificial Intelligence
Source: Nat Commun. 2022 Oct 3;13:5821. doi: 10.1038/s41467-022-33417-3 (PMC9528860; doi:10.1038/s41467-022-33417-3)
Supplement: Supplementary file 1 — Supplementary Information [file 41467_2022_33417_MOESM1_ESM.pdf]

Supplementary Information for

Public attitudes value interpretability but prioritize accuracy in Artificial Intelligence

Anne-Marie Nussberger[1*], Lan Luo[2], L. Elisa Celis[3], Molly J. Crockett[4*]

[1] Center for Humans and Machines, Max Planck Institute for Human Development, Berlin, Germany
[2] Department of Marketing, Columbia Business School
[3] Department of Statistics and Data Science, Yale University
[4] Department of Psychology, Princeton University

* Corresponding authors: Anne-Marie Nussberger (nussberger@mpib-berlin.mpg.de; ORCID: 0000-0002-1805-9399); Molly J. Crockett (mc5121@princeton.edu; OCRID: 0000-0001-8800-410X)

Supplementary Methods

Participants

Study 3D (only in SI)

In line with our pre-registration, we recruited 1,614 US participants via Prolific (data collected 15-21/01/2021), using the platform's feature for collecting representative samples that match census data in terms of age by sex by ethnic group proportions. We excluded 22 duplicate cases, 87 participants who did not finish the survey (progress ≤ 85%), and 87 participants who failed a simple comprehension check on two attempts, leaving a final sample of N = 1,418. The final sample included 687 males, 713 females, 13 nonbinary, and 5 "prefer not to say" with an average age of 44.61 (SD = 16.38, SE = 0.44). 5 of the participants' highest education was less than high school; 135, high school; 334, some college; 136, a two-year degree; 499, a four-year degree; 309, a postgrad or another professional degree. The mean income bracket was between $35,001 and $50,000. Most participants (691) had no formal education in computer science; 261 had some programming experience; 337 took a college-level course; 76 held an undergraduate degree; and 53 held a graduate degree in computer science.

Study 4 (only in SI)

We recruited 249 participants via MTurk (data collected 11-12/12/2019). The final sample included 163 males, 85 females, and 1 "other" with an average age of 35.57 (SD = 9.62, SE = 0.61). 2 of the participants' highest education was less than high school; 28, high school; 50, some college; 24, two-year degree; 118, four-year degree; and 27, postgrad or other professional degree. The mean income bracket was between $35,001 and $50,000. 112 of the participants had no computer science knowledge; 47, some experience; 46, a college degree; 22, an undergraduate degree; and 22, a graduate degree.

Motivation and task

Study 3D (only in SI)

Study 3B used a within-subjects design that manipulated stakes and scarcity of various AI applications. The main effects for stakes and scarcity on people's preferences for accuracy and interpretability in AI demonstrated in this study replicated in a between-subjects design deployed in Study 3C, where each participant was presented with only one combination of stakes and scarcity. However, smaller effect sizes in Study 3C relative to Study 3B might point towards saliency of variation in the attributes playing at least some role in the effects of stakes and scarcity. Instructions in Study 3C still mentioned that there is such variation (e.g., "Vaccine supply can be abundant or limited. […] Some vaccines protect humans against mild variants of the flu, while other vaccines protect humans against deadly variants of the flu") before specifying the specific combination for a given participants (e.g., "In this case, vaccine supply is limited. The vaccine protects against a deadly variant of the flu"). Study 3D completely omitted information about possible variation in stakes and scarcity. Instead, it only instructed participants about the given case at hand ("vaccine supply is very limited. […] This particular vaccine protects against a deadly variant of the flu"; see SI Methods, Materials Study 3D). These instructions thus eliminated any saliency of possible variation in stakes and scarcity and their potential to enhance participants' sensitivity to such variations[1,2].

Study 4 (only in SI)

Taken together, Studies 1A to 3C provide evidence that people value interpretability in AI, although they prioritize accuracy over interpretability when these features trade off with one another. Moreover, stakes and scarcity drove preferences for accuracy over interpretability in the same direction as their impact on preferences for interpretability alone. In Study 4, we sought to replicate and extend these findings by examining how stakes and scarcity affect preferences for interpretability and accuracy independently as well as in tandem, and whether these effects are robust across different stakeholder perspectives. In particular, past research has often characterised interpretability in AI as means to the higher end of justifying machine-generated decisions[3,4]. Justifying a decision is conceivably more important from the perspective of a responsible *agent*, who oversees the decision, relative to the perspective of a *patient*, who

is affected by the decision. This might produce enhanced interpretability-requirements by agents relative to patients. To test this hypothesis, participants (final N = 266; American sample recruited from MTurk) first indicated their preferences for interpretability at the expense of accuracy (as in Studies 3B-3C) and subsequently indicated their preferences for interpretability and accuracy separately. To test for the effect of different stakeholder perspectives, we additionally varied between-subjects whether participants evaluated the AI application from the perspective of a responsible *agent* who manages the AI application, versus a *patient* who is directly impacted by the AI's decision. The applications descriptions were essentially the same as in Studies 3A and 3B, although we added sentences that specified the relevant perspective (see SI Methods, Materials Study 4).

Supplementary Results

Study 1A

Pre- & post-task support for ML. Participants answered the question "How much do you oppose or support the use of AI" both before and after completing the main task on a 5-point rating scale ranging from "strongly oppose" to "strongly support". Responses did not differ between pre- and post-task measurement (b = 0.01, p = .909) nor across *recommend* and *decide* conditions (b = 0.16, p = .113). Summarizing across times of measurement, participants overall supported the use of AI, averaging at 3.90 which exceeded the scale-midpoint, t(339) = 17.56, p < .001, d = .95 (see *Table SI 1*).

	Dependent variable:
	Support for ML
Post-Task	0.012
	(0.102)
Decide Condition	0.163
	(0.103)
Constant	3.809***
	(0.090)
Observations	340
Adjusted R^2	0.002
Note:	*p<0.05; **p<0.01; ***p<0.001

Table SI 1. Pre- and Post-Task Support for ML (Study 1A). P-values are determined by a two-sided t-test with no adjustment for multiple comparisons: $p_{Post\text{-}Task}$ = .909; $p_{Decide\ Condition}$ = .113; $p_{Constant}$ < .001. Standard errors are included in parentheses.

Intuitions about ability to explain ML. Before completing the main task, participants subsequently answered the question "To what extent can [people without/experts with] training in computer science explain how an AI reaches certain predictions, recommendations, or decisions in certain cases? By "explain" we mean that they can explain how an AI reaches a certain prediction, recommendation, or decision in non-technical terms" on a 5-point rating scale ranging from "can explain not at all" to "can explain fully". Participants indicated that experts with training in computer science could explain AI to a significantly larger extent (M = 4.40) than people without training in computer science, M = 2.36; t(169) = -24.18, p < .001, d = -1.85.

Sphere as explanatory factor for demand in interpretability. One might suspect that applications situated in the public sphere (e.g., 'military weapons operations') elicit higher demand for interpretability than applications largely situated in the private sphere (e.g., 'organizing pictures')[55,56]. Entering a hand-coded predictor categorizing applications as private or public into an ordinal regression analysis that predicted participants' ratings for the importance of interpretability suggested this might be the case: applications in the public sphere elicited higher demand for interpretability, $\hat{\beta}$ = 0.73, p < .001, 95% CI [0.59, 0.88]. One important limitation of our exploration of sphere as an explanatory factor for demand in interpretability is

that our posthoc categorization yielded a disbalanced distribution of applications across the two categories public (15 applications) and private (8 applications) and that there were four applications that we omitted from the analyses because they were too ambiguous (*viz.* cyber security, hiring decisions, insurance pricing, improving food flavour). Nonetheless, we think that our exploratory results indicate that this might be a potent avenue for future research that deserves a series of experiments on its own.

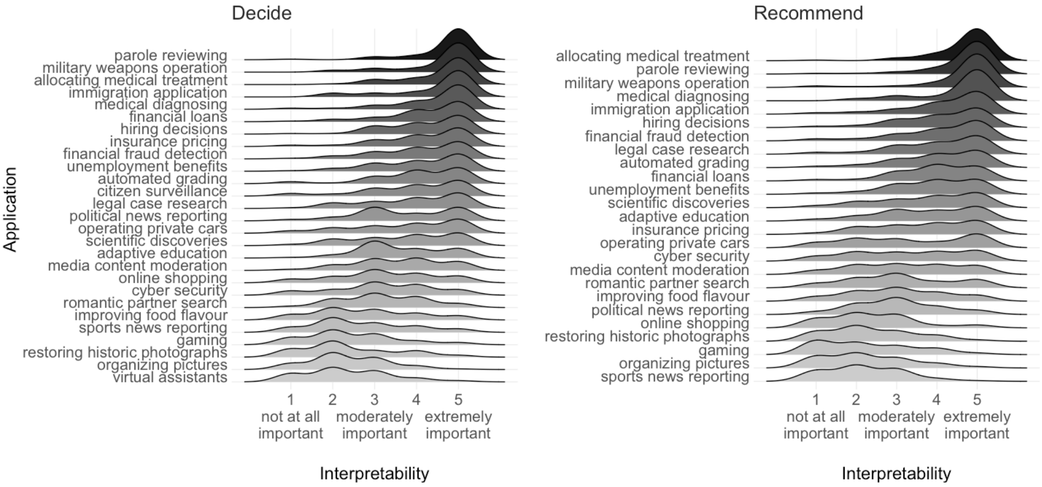

Figure SI 1. Joyplots visualizing the distributions of interpretability ratings for decide and recommend versions separately. Participants responded to the question “how important is it that the AI in this application is explainable, even if it performs accurately?” on a 5-point rating scale (1 = not at all important, 5 = extremely important).

Study 1B

Main effects. We observed a significant main effect for stakes, $F(2, 7479) = 1139.70$, $p < .001$, $\eta^2 = 0.23$, 95% CI [0.22, 0.25]. Relative to applications involving low stakes, demand for interpretability was stronger for applications involving high ($b = 1.49$, $p < .001$, 95% CI [1.43, 1.55]) or medium stakes ($b = 0.93$, $p < .001$, 95% CI [0.86, 1.00]), and it was stronger amidst high than medium stakes, $b = 0.56$, $p < .001$, 95% CI [0.50, 0.62]. Similarly, a significant main effect for scarcity ($F(1, 7480) = 313.02$, $p < .001$, $\eta^2 = 0.04$, 95% CI [0.03, 0.05]) indicated increased demand for interpretability in applications potentially allocating scarce resources, relative to those that did not, $b = 0.58$, $p < .001$, 95% CI [0.51, 0.64].

Pre- & post-task support for ML. Responses did not differ between pre- and post-task measurement ($b = 0.09$, $p = .244$). Summarizing across times of measurement, participants overall supported the use of AI, averaging at 3.80 which exceeded the scale-midpoint, $t(515) = 20.92$, $p < .001$, $d = .92$ (see *Table SI 2*).

	Dependent variable:
	Support for ML
Post-Task	0.089
	(0.076)
Constant	3.752***
	(0.054)
Observations	516
Adjusted R^2	0.001
Note:	*p<0.05; **p<0.01; ***p<0.001

Table SI 2. Pre- and Post-Task Support for ML (Study 1B). P-values are determined by a two-sided t-test with no adjustment for multiple comparisons: $p_{Post\text{-}Task}$ = .244; $p_{Constant}$ < .001. Standard errors are included in parentheses.

Study 1C

Main effects. We observed a significant main effect for stakes, *F*(2, 7131) = 1097.00, *p* < .001, η^2 = 0.24, 95% CI [0.22, 0.25]. Relative to applications involving low stakes, demand for interpretability was stronger for applications involving high (*b* = 1.52, *p* < .001, 95% CI [1.45, 1.58]) or medium stakes (*b* = 0.95, *p* < .001, 95% CI [0.88, 1.02]), and it was stronger amidst high than medium stakes, *b* = 0.57, *p* < .001, 95% CI [0.50, 0.63]. Similarly, a significant main effect for scarcity (*F*(1, 7132) = 271.50, *p* < .001, η^2 = 0.04, 95% CI [0.03, 0.04]) indicated increased demand for interpretability in applications potentially allocating scarce resources, relative to those that did not, *b* = 0.56, *p* < .001, 95% CI [0.50, 0.63].

Pre- & post-task support for ML. Responses did not differ between pre- and post-task measurement (*b* = 0.14, *p* = .053). Summarizing across times of measurement, participants overall supported the use of AI, averaging at 3.75 which exceeded the scale-midpoint, *t*(491) = 21.50, *p* < .001, *d* = .97 (see *Table SI 3*).

	Dependent variable:
	Support for ML
Post-Task	0.135
	(0.070)
Constant	3.683***
	(0.049)
Observations	492
Adjusted R^2	0.006
Note:	*p<0.05; **p<0.01; ***p<0.001

Table SI 3. Pre- and Post-Task Support for ML (Study 1C). P-values are determined by a two-sided t-test with no adjustment for multiple comparisons: $p_{Post\text{-}Task}$ = .053; $p_{Constant}$ < .001. Standard errors are included in parentheses.

Study 2

Pre- & post-task support for ML. Responses did not differ between pre- and post-task measurement (*b* = 0.04, *p* = .766). Summarizing across times of measurement, participants overall supported the use of AI, averaging at 3.99 which exceeded the scale-midpoint, *t*(167) = 14.79, *p* < .001, *d* = 1.14 (see *Table SI 4*).

	Dependent variable:
	Support for ML
Post-Task	0.040
	(0.135)
Constant	3.974***
	(0.095)
Observations	168
Adjusted R^2	−0.005
Note:	*p<0.05; **p<0.01; ***p<0.001

Table SI 4. Pre- and Post-Task Support for ML (Study 2). P-values are determined by a two-sided t-test with no adjustment for multiple comparisons: $p_{Post\text{-}Task}$ = .766; $p_{Constant}$ < .001. Standard errors are included in parentheses.

Model with general controls. Main effects for *stakes* and *scarcity* were robust to controlling for gender, age, education, income, pre-task support for ML, awareness of AI bias, and computer science knowledge (see *Table SI 5*).

	Dependent variable:
	Demand for Interpretability
Stakes	0.847***
	(0.064)
Scarcity	0.485***
	(0.064)
Stakes x Scarcity	−0.028
	(0.091)
Gender	0.236
	(0.143)
Age	−0.010
	(0.006)
Education	0.059
	(0.060)
Income	0.015
	(0.036)
Pre-Task Support for ML	0.096
	(0.077)
Heard about Bias in AI	−0.078
	(0.178)
CS (Some Programming Experience)	−0.244
	(0.293)
CS (College Course)	−0.018
	(0.305)
CS (Undergraduate Degree)	−0.002
	(0.301)
CS (Graduate Degree)	0.376
	(0.395)
Constant	2.855***
	(0.606)
Observations	1,640
Log Likelihood	−2,278.812
Akaike Inf. Crit.	4,591.624
Bayesian Inf. Crit.	4,683.466
Note:	*p<0.05; **p<0.01; ***p<0.001

Table SI 5. Linear Mixed Model: Effect of Stakes, Scarcity on Demand for Interpretability with Controls (Study 2). P-values are determined by a two-sided t-test (using Satterthwaite's method for denominator degrees of freedom) with no adjustment for multiple comparisons: p_{Stakes} < .001; $p_{Scarcity}$ < .001; $p_{Stakes\ x\ Scarcity}$ = .758; p_{Gender} = .100; p_{Age} = .067; $p_{Education}$ = .323; p_{Income} = .685; $p_{Pre\text{-}Task\ Support\ for\ ML}$ = .214; $p_{Heard\ about\ Bias\ in\ AI}$ = .663; $p_{CS\ (Some)}$ = .405; $p_{CS\ (College)}$ = .952; $p_{CS\ (Undergrad)}$ = .994; $p_{CS\ (Grad)}$ = .341; $p_{Constant}$ < .001. Standard errors are included in parentheses.

Separate models for each application (see *Table SI 6*).

	Dependent variable:				
	Demand for Interpretability				
	Vaccination	First Responders	Insurance	Hiring	Standby Seats
	(1)	(2)	(3)	(4)	(5)
Stakes	1.033***	0.943***	0.937***	0.757***	0.578***
	(0.134)	(0.116)	(0.117)	(0.131)	(0.121)
Scarcity	0.862***	0.620***	0.728***	0.082	0.137
	(0.134)	(0.116)	(0.117)	(0.131)	(0.121)
Stakes x Scarcity	−0.223	0.026	−0.106	0.091	0.072
	(0.189)	(0.164)	(0.165)	(0.186)	(0.170)
Constant	2.897***	2.993***	3.010***	3.002***	3.072***
	(0.116)	(0.110)	(0.109)	(0.119)	(0.132)
Observations	336	336	336	336	336
Log Likelihood	−474.976	−441.503	−440.080	−475.722	−472.732
Akaike Inf. Crit.	961.953	895.006	892.159	963.444	957.465
Bayesian Inf. Crit.	984.855	917.908	915.062	986.346	980.368
Note:					*p<0.05; **p<0.01; ***p<0.001

Table SI 6. Linear Mixed Model by Application: Effect of Stakes, Scarcity on Demand for Interpretability (Study 2). P-values are determined by a two-sided t-test (using Satterthwaite's method for denominator degrees of freedom) with no adjustment for multiple comparisons: for Vaccination, $p_{Stakes} < .001$; $p_{Scarcity} < .001$; $p_{Stakes\ x\ Scarcity} = .239$; $p_{Constant} < .001$; for First Responders, $p_{Stakes} < .001$; $p_{Scarcity} < .001$; $p_{Stakes\ x\ Scarcity} = .874$; $p_{Constant} < .001$; for Insurance, $p_{Stakes} < .001$; $p_{Scarcity} < .001$; $p_{Stakes\ x\ Scarcity} = .519$; $p_{Constant} < .001$; for Hiring, $p_{Stakes} < .001$; $p_{Scarcity} = .535$; $p_{Stakes\ x\ Scarcity} = .625$; $p_{Constant} < .001$; for Standby Seats, $p_{Stakes} < .001$; $p_{Scarcity} = .257$; $p_{Stakes\ x\ Scarcity} = .672$; $p_{Constant} < .001$. Standard errors are included in parentheses.

Study 3A

Pre- & post-task support for ML. Responses did not differ between pre- and post-task measurement ($b = -0.05$, $p = .573$). Summarizing across times of measurement, participants overall supported the use of AI, averaging at 3.74 which exceeded the scale-midpoint, $t(521) = 18.66$, $p < .001$, $d = 0.82$ (see *Table SI 7*).

	Dependent variable:
	Support for ML
Post-Task	−0.045
	(0.079)
Constant	3.759***
	(0.056)
Observations	522
Adjusted R^2	−0.001
Note:	*p<0.05; **p<0.01; ***p<0.001

Table SI 7. Pre- and Post-Task Support for ML (Study 3A). P-values are determined by a two-sided t-test with no adjustment for multiple comparisons: $p_{Post\text{-}Task} = .573$; $p_{Constant} < .001$. Standard errors are included in parentheses.

Model with general controls (see *Table SI 8*).

	Dependent variable:
	Demand for Interpretability
70% Accuracy	−0.033
	(0.040)
80% Accuracy	−0.098*
	(0.040)
90% Accuracy	−0.112**
	(0.040)
Low to High Accuracy Ordering	0.015
	(0.081)
Gender	−0.143
	(0.085)
Age	−0.002
	(0.003)
Education	−0.033
	(0.036)
Income	−0.025
	(0.016)
Pre-Task Support for ML	−0.066
	(0.046)
Heard about Bias in AI	0.233*
	(0.099)
CS (College Course)	−0.005
	(0.093)
CS (Undergraduate Degree)	0.011
	(0.170)
CS (Graduate Degree)	−0.346
	(0.242)
Constant	4.216***
	(0.326)
Observations	5,080
Log Likelihood	−7,484.724
Akaike Inf. Crit.	15,003.450
Bayesian Inf. Crit.	15,114.510
Note:	*p<0.05; **p<0.01; ***p<0.001

Table SI 8. Linear Mixed Model: Effect of Accuracy Levels on Demand for Interpretability with General Controls (Study 3A). P-values are determined by a two-sided t-test (using Satterthwaite's method for denominator degrees of freedom) with no adjustment for multiple comparisons: $p_{70\%\ Accuracy}$ = .399; $p_{80\%\ Accuracy}$ = .014; $p_{90\%\ Accuracy}$ = .005; $p_{Low\ to\ High\ Accuracy\ Ordering}$ = .855; p_{Gender} = .094; p_{Age} = .506; $p_{Education}$ = .350; p_{Income} = .132; $p_{Pre\text{-}Task\ Support\ for\ ML}$ = .153; $p_{Heard\ about\ Bias\ in\ AI}$ = .019; $p_{CS\ (College)}$ = .953; $p_{CS\ (Undergrad)}$ = .949; $p_{CS\ (Grad)}$ = .154; $p_{Constant}$ < .001. Standard errors are included in parentheses.

Model with explanatory controls (see *Table SI 9*).

	Dependent variable:
	Demand for Interpretability
70% Accuracy	−0.032
	(0.038)
80% Accuracy	−0.097*
	(0.038)
90% Accuracy	−0.114**
	(0.038)
Human Accuracy	0.048*
	(0.020)
Reversibility	−0.054***
	(0.013)
Human Expertise	0.267***
	(0.019)
Personal Affectedness	0.116***
	(0.014)
No. People Affected	−0.015
	(0.009)
Constant	2.511***
	(0.149)
Observations	5,212
Log Likelihood	−7,499.905
Akaike Inf. Crit.	15,023.810
Bayesian Inf. Crit.	15,102.510
Note:	*p<0.05; **p<0.01; ***p<0.001

Table SI 9. Linear Mixed Model: Effect of Accuracy Levels on Demand for Interpretability with Explanatory Controls (Study 3A). P-values are determined by a two-sided t-test (using Satterthwaite's method for denominator degrees of freedom) with no adjustment for multiple comparisons: $p_{70\%\ Accuracy}$ = .399; $p_{80\%\ Accuracy}$ = .011; $p_{90\%\ Accuracy}$ = .003; $p_{Human\ Accuracy}$ = .016; $p_{Reversibility}$ < .001; $p_{Human\ Expertise}$ < .001; $p_{Personal\ Affectedness}$ < .001; $p_{No.\ People\ Affected}$ = .103; $p_{Constant}$ < .001. Standard errors are included in parentheses.

Study 3B

Pre- & post-task support for ML. Responses did not differ between pre- and post-task measurement (*b* = -0.04, *p* = .719). Summarizing across times of measurement, participants overall supported the use of AI, averaging at 4.02 which exceeded the scale-midpoint, *t*(223) = 17.31, *p* < .001, *d* = 1.16 (see *Table SI 10*).

	Dependent variable:
	Support for ML
Post-Task	−0.042
	(0.118)
Constant	4.040***
	(0.083)
Observations	224
Adjusted R^2	−0.004
Note:	*p<0.05; **p<0.01; ***p<0.001

Table SI 10. Pre- and Post-Task Support for ML (Study 3B). P-values are determined by a two-sided t-test with no adjustment for multiple comparisons: $p_{Post\text{-}Task}$ = .719; $p_{Constant}$ < .001. Standard errors are included in parentheses.

Model with general controls. Main effects for *stakes* and *scarcity* were robust to controlling for gender, age, education, income, pre-task support for ML, awareness of AI bias, and computer science knowledge (see *Table SI 11*).

	Dependent variable:
	Trade-Off Preferences
Stakes	−0.426***
	(0.072)
Scarcity	−0.306***
	(0.072)
Stakes x Scarcity	0.107
	(0.101)
Gender	0.004
	(0.151)
Age	−0.007
	(0.006)
Education	0.061
	(0.060)
Income	−0.010
	(0.033)
Pre-Task Support for ML	−0.105
	(0.075)
Heard about Bias in AI	0.049
	(0.178)
CS (Some Programming Experience)	−0.205
	(0.183)
CS (College Course)	−0.140
	(0.177)
CS (Undergraduate Degree)	0.031
	(0.284)
CS (Graduate Degree)	0.315
	(0.267)
Constant	0.506
	(0.498)
Observations	2,220
Log Likelihood	−3,644.818
Akaike Inf. Crit.	7,323.636
Bayesian Inf. Crit.	7,420.626
Note:	*p<0.05; **p<0.01; ***p<0.001

Table SI 11. Linear Mixed Model: Effect of Stakes, Scarcity on Trade-Off Preferences with General Controls (Study 3B). P-values are determined by a two-sided t-test (using Satterthwaite's method for denominator degrees of freedom) with no adjustment for multiple comparisons: $p_{Stakes} < .001$; $p_{Scarcity} < .001$; $p_{Stakes\ x\ Scarcity} = .290$; $p_{Gender} = .977$; $p_{Age} = .278$; $p_{Education} = .309$; $p_{Income} = .772$; $p_{Pre\text{-}Task\ Support\ for\ ML} = .159$; $p_{Heard\ about\ Bias\ in\ AI} = .784$; $p_{CS\ (Some)} = .263$; $p_{CS\ (College)} = .429$; $p_{CS\ (Undergrad)} = .914$; $p_{CS\ (Grad)} = .239$; $p_{Constant} = .310$. Standard errors are included in parentheses.

Model with explanatory controls. To further explore explanatory candidates, we asked participants the following additional questions once they had completed the main task; they provided answers for general versions of each application:

Reversibility: “How reversible is a decision performed by the AI?”
Human expertise: “What level of expertise would be required for a human to perform the decision instead of the AI?”
Personal affectedness: “How likely is it that such a decision would affect you personally?”
Number of people affected: “How many people will be affected by the decisions performed by the AI?”

When we added these explanatory variables to our mixed effects model predicting preferences over accuracy versus interpretability, main effects for stakes and scarcity remained significant while we also observed a significant main effect for *human expertise,* whereby demand for accuracy over explainability was more pronounced in those applications that they considered to require a high level of human expertise (see *Table SI 12*).

	Dependent variable:
	Trade-Off Preferences
Stakes	−0.468***
	(0.079)
Scarcity	−0.337***
	(0.079)
Stakes x Scarcity	0.163
	(0.112)
Reversibility	−0.011
	(0.025)
Human Expertise	−0.235***
	(0.033)
Personal Affectedness	0.048
	(0.029)
No. People Affected	−0.020
	(0.017)
Constant	0.831***
	(0.173)
Observations	1,792
Log Likelihood	−2,944.838
Akaike Inf. Crit.	5,911.676
Bayesian Inf. Crit.	5,972.078
Note:	*p<0.05; **p<0.01; ***p<0.001

Table SI 12. Linear Mixed Model: Effect of Stakes, Scarcity on Trade-off Preferences with Explanatory Controls (Study 3B). P-values are determined by a two-sided t-test (using Satterthwaite's method for denominator degrees of freedom) with no adjustment for multiple comparisons: $p_{Stakes} < .001$; $p_{Scarcity} < .001$; $p_{Stakes\ x\ Scarcity} = .145$; $p_{Reversibility} = .662$; $p_{Human\ Expertise} < .001$; $p_{Personal\ Affectedness} = .104$; $p_{No.\ People\ Affected} = .237$; $p_{Constant} < .001$. Standard errors are included in parentheses.

Study 3C

Pre- & post-task support for ML. Responses did not differ between pre- and post-task measurement ($b = 0.05$, $p = .143$). Summarizing across times of measurement, participants overall supported the use of AI, averaging at 3.86 which exceeded the scale-midpoint, $t(2686) = 54.98$, $p < .001$, $d = 1.06$ (see *Table SI 13*).

	Dependent variable:
	Support for ML
Post-Task	0.046
	(0.031)
Constant	3.835***
	(0.022)
Observations	2,687
Adjusted R^2	0.0004
Note:	*p<0.05; **p<0.01; ***p<0.001

Table SI 13. Pre- and Post-Task Support for ML (Study 3C). P-values are determined by a two-sided t-test with no adjustment for multiple comparisons: $p_{Post\text{-}Task} = .143$; $p_{Constant} < .001$. Standard errors are included in parentheses.

Model with general controls. Main effects for *stakes* and *scarcity* were robust to controlling for gender, age, education, income, pre-task support for ML, awareness of AI bias, and computer science knowledge (see *Table SI 14*).

	Dependent variable:
	Trade-Off Preferences
Stakes	-0.300^{***}
	(0.057)
Scarcity	-0.165^{**}
	(0.056)
Stakes x Scarcity	0.106
	(0.080)
Gender	0.067
	(0.045)
Age	-0.005^{**}
	(0.001)
Education	0.038^{**}
	(0.014)
Income	0.013
	(0.008)
Pre-Task Support for ML	−0.017
	(0.026)
Heard about Bias in AI	0.009
	(0.050)
CS (Some Programming Experience)	0.072
	(0.062)
CS (College Course)	−0.009
	(0.050)
CS (Undergraduate Degree)	−0.116
	(0.116)
CS (Graduate Degree)	0.464^{***}
	(0.088)
Constant	−0.176
	(0.201)
Observations	6,610
Log Likelihood	−11,211.870
Akaike Inf. Crit.	22,457.740
Bayesian Inf. Crit.	22,573.280
Note:	*p<0.05; **p<0.01; ***p<0.001

Table SI 14. Linear Mixed Model: Effect of Stakes, Scarcity on Trade-Off Preferences with General Controls (Study 3C). P-values are determined by a two-sided t-test (using Satterthwaite's method for denominator degrees of freedom) with no adjustment for multiple comparisons: $p_{Stakes} < .001$; $p_{Scarcity} = .004$; $p_{Stakes\ x\ Scarcity} = .184$; $p_{Gender} = .134$; $p_{Age} = .002$; $p_{Education} = .007$; $p_{Income} = .099$; $p_{Pre\text{-}Task\ Support\ for\ ML} = .517$; $p_{Heard\ about\ Bias\ in\ AI} = .861$; $p_{CS\ (Some)} = .250$; $p_{CS\ (College)} = .857$; $p_{CS\ (Undergrad)} = .318$; $p_{CS\ (Grad)} < .001$; $p_{Constant} = .381$. Standard errors are included in parentheses.

Model with explanatory controls. When we added explanatory variables to our mixed effects model predicting preferences over accuracy versus interpretability, main effects for stakes and scarcity remained significant. We observed a significant main effect for *reversibility,* whereby demand for accuracy over explainability was less pronounced in applications for which they considered the AI's decisions to be reversible. There was also a significant main effect for *human expertise,* whereby demand for accuracy over explainability was more pronounced in applications they considered to require a high level of human expertise (see *Table SI 15*).

	Dependent variable:
	Trade-Off Preferences
Stakes	−0.271***
	(0.057)
Scarcity	−0.140*
	(0.057)
Stakes x Scarcity	0.067
	(0.081)
Reversibility	0.078***
	(0.014)
Human Expertise	−0.106***
	(0.019)
Personal Affectedness	0.023
	(0.015)
No. People Affected	−0.011
	(0.008)
Constant	0.005
	(0.152)
Observations	6,707
Log Likelihood	−11,373.210
Akaike Inf. Crit.	22,768.420
Bayesian Inf. Crit.	22,843.340
Note:	*p<0.05; **p<0.01; ***p<0.001

Table SI 15. Linear Mixed Model: Effect of Stakes, Scarcity on Trade-off Preferences with Explanatory Controls (Study 3C). P-values are determined by a two-sided t-test (using Satterthwaite's method for denominator degrees of freedom) with no adjustment for multiple comparisons: $p_{Stakes} < .001$; $p_{Scarcity} = .015$; $p_{Stakes\ x\ Scarcity} = .409$; $p_{Reversibility}$ = < .001; $p_{Human\ Expertise} < .001$; $p_{Personal\ Affectedness} = .119$; $p_{No.\ People\ Affected} = .175$; $p_{Constant} = .973$. Standard errors are included in parentheses.

Study 3D (only in SI)

Results. Again, we coded participants' responses such that positive values represented a preference for interpretability over accuracy and negative values indicated a preference for accuracy over interpretability. In line with our findings from Studies 3A and 3B, we observed an overall preference for accuracy over interpretability, signified by a negative average of *M* = -0.36 that differed significantly from the indifference point, *t*(7,087) = -22.12, *p* < .001, 95% CI [-0.39, -0.32].

Next, we ran a linear mixed effects model predicting participants' tradeoff preferences, with *stakes*, *scarcity*, and their interaction entered as fixed effects while we entered *subject* and *application* as random intercept effects. Using type II Wald chi-square tests to probe the fixed effects' significance, we observed a significant main effect for *stakes* ($\chi^2(1) = 25.56$, *p* < .001; *b* = -0.15, *p* = .004, *d* = 0.03, 95% CI [-0.01, 0.08]), while the effect for *scarcity* was not significant ($\chi^2(1) = 2.22$, *p* = .136), nor was the interaction between *stakes* and *scarcity*, $\chi^2(1) = 0.95$, *p* = .329.

As we discuss in our main manuscript, with the accelerating spread of AI, people will be increasingly likely to encounter variations of stakes and scarcity within and across AI-applications in the real-world. This will arguably enhance their sensitivity to stakes and scarcity present in a given AI application and foster the formation of more systematic and stable preferences over accuracy and interpretability in AI[2]. However, the results from Studies 3B and

3C in particular suggest that the observed effects of stakes and scarcity may partially hinge on the salience of variation in the two attributes.

Pre- & post-task support for ML. Responses did not differ between pre- and post-task measurement (b = -0.01, p = .745). Summarizing across times of measurement, participants overall supported the use of AI, averaging at 3.76 which exceeded the scale-midpoint, t(2835) = 48.55, p < .001, d = 0.91 (see *table SI 16*).

	Dependent variable:
	Support for ML
Post-Task	−0.010
	(0.031)
Constant	3.768***
	(0.022)
Observations	2,836
Adjusted R^2	−0.0003
Note:	*p<0.05; **p<0.01; ***p<0.001

Table SI 16. Pre- and Post-Task Support for ML (Study 3D). P-values are determined by a two-sided t-test with no adjustment for multiple comparisons: $p_{Post\text{-}Task}$ = .745; $p_{Constant}$ < .001. Standard errors are included in parentheses.

Model with general controls. Main effects for *stakes* but not *scarcity* were robust to controlling for gender, age, education, income, pre-task support for ML, awareness of AI bias, and computer science knowledge (see *Table SI 17*).

	Dependent variable:
	Trade-Off Preferences
Stakes	−0.151**
	(0.052)
Scarcity	−0.019
	(0.051)
Stakes x Scarcity	−0.082
	(0.073)
Gender	0.004
	(0.039)
Age	−0.002
	(0.001)
Education	0.028*
	(0.014)
Income	0.017*
	(0.007)
Pre-Task Support for ML	−0.059**
	(0.023)
Heard about Bias in AI	0.098*
	(0.042)
CS (Some Programming Experience)	0.108*
	(0.053)
CS (College Course)	0.016
	(0.046)
CS (Undergraduate Degree)	−0.039
	(0.087)
CS (Graduate Degree)	0.185
	(0.102)
Constant	−0.309
	(0.226)
Observations	6,978
Log Likelihood	−11,681.840
Akaike Inf. Crit.	23,397.690
Bayesian Inf. Crit.	23,514.150
Note:	*p<0.05; **p<0.01; ***p<0.001

Table SI 17. Linear Mixed Model: Effect of Stakes, Scarcity on Trade-Off Preferences with General Controls (Study 3D). P-values are determined by a two-sided t-test (using Satterthwaite's method for denominator degrees of freedom) with no adjustment for multiple comparisons: p_{Stakes} = .004; $p_{Scarcity}$ = .712; $p_{Stakes\ x\ Scarcity}$ = .262; p_{Gender} = .010; p_{Age} = .022; $p_{Education}$ = .726; p_{Income} = .071; $p_{Pre\text{-}Task\ Support\ for\ ML}$ = .041; $p_{Heard\ about\ Bias\ in\ AI}$ = .657; $p_{CS\ (Some)}$ = .909; $p_{CS\ (College)}$ = .134; $p_{CS\ (Undergrad)}$ = .041; $p_{CS\ (Grad)}$ = .019; $p_{Constant}$ = .172. Standard errors are included in parentheses.

Model with explanatory controls. When we added explanatory variables to our mixed effects model predicting preferences over accuracy versus interpretability, main effects for stakes but not scarcity remained significant. We observed a significant main effect for *reversibility,* whereby demand for accuracy over explainability was less pronounced in applications for which they considered the AI's decisions to be reversible. There was also a significant main effect for *human expertise,* whereby demand for accuracy over explainability was more pronounced in applications they considered to require a high level of human expertise (see *Table SI 18*).

	Dependent variable:
	Trade-Off Preferences
Stakes	−0.140**
	(0.052)
Scarcity	−0.009
	(0.052)
Stakes x Scarcity	−0.080
	(0.073)
Reversibility	0.086***
	(0.013)
Human Expertise	−0.140***
	(0.017)
Personal Affectedness	0.006
	(0.013)
No. People Affected	−0.013
	(0.008)
Constant	0.026
	(0.167)
Observations	7,070
Log Likelihood	−11,778.700
Akaike Inf. Crit.	23,579.400
Bayesian Inf. Crit.	23,654.900
Note:	*p<0.05; **p<0.01; ***p<0.001

Table SI 18. Linear Mixed Model: Effect of Stakes, Scarcity on Trade-off Preferences with Explanatory Controls (Study 3D). P-values are determined by a two-sided t-test (using Satterthwaite's method for denominator degrees of freedom) with no adjustment for multiple comparisons: p_{Stakes} = .007; $p_{Scarcity}$ = .869; $p_{Stakes\ x\ Scarcity}$ = .276; $p_{Reversibility}$ < .001; $p_{Human\ Expertise}$ < .001; $p_{Personal\ Affectedness}$ = .662; $p_{No.\ People\ Affected}$ = .119; $p_{Constant}$ = .879. Standard errors are included in parentheses.

Study 4 (only in SI)

Results. First, we tested the main effects of stakes and scarcity on preferences for interpretability relative to accuracy when the two features traded off against one another. Again, participants overall prioritized accuracy over interpretability, as indicated by a mean tradeoff rating of *M* = -0.36, which differed significantly from the indifference point of 0, *t*(4,978) = -18.28, *p* < .001, 95% CI [-0.40, -0.32]. Similarly, we replicated main effects for *stakes* (χ^2(1) = 218.27, *p* < .001) and *scarcity* (χ^2(1) = 61.70, *p* < .001) on tradeoff preferences, which were not qualified by an interaction (χ^2(1) = 0.18, *p* = .676). These main effects followed the previously established pattern whereby participants' preference for accuracy was more pronounced for high- relative to low-stakes cases (*b* = -0.51, *p* < .001, *d* = 0.15, 95% CI [0.10, 0.20]) and for cases characterized by high relative to low scarcity, *b* = -0.28, *p* < .001, *d* = 0.08, 95% CI [0.03, 0.13]. These results generalized across *patient* and *agent* perspectives: when we added a corresponding perspective-variable and interaction effects to the mixed effects model, main

effects for *stakes* ($\chi^2(1) = 218.34$, $p < .001$) and *scarcity* ($\chi^2(1) = 61.72$, $p < .001$) remained significant, while there was no main effect for *perspective* ($\chi^2(1) = 0.03$, $p = .861$) nor any significant two- or three-way-interactions between *perspective* and the other two predictors (*p*s ≥ .203).

Next, we examined the effects of stakes and scarcity on preferences for interpretability and accuracy as measured independently. We observed a significant overall demand for interpretability ($M = 3.29$), which exceeded the "moderately important" scale-midpoint, $t(4{,}978) = 17.31$, $p < .001$, 95% CI [3.26, 3.33]. Similarly, we observed a significant demand for accuracy ($M = 3.82$), which also exceeded the "moderately important" scale-midpoint, $t(4{,}979) = 50.21$, $p < .001$, 95% CI [3.79, 3.85], and the demand for accuracy was significantly higher than the demand for interpretability, $|t(9{,}941)| = 22.29$, $p < .001$, |95% CI| [0.57, 0.48], consistent with the results obtained from the tradeoff measure. We again replicated the main effects for *stakes* and *scarcity* on both separate measures (regressing on interpretability: *stakes:* $\chi^2(1) = 14.71$, $p < .001$; *scarcity:* $\chi^2(1) = 8.97$, $p = .003$; regressing on accuracy: *stakes:* $\chi^2(1) = 391.61$, $p < .001$; *scarcity:* $\chi^2(1) = 98.97$, $p < .001$). As with the tradeoff measure, there was no main effect of stakeholder perspective on either preferences for interpretability or accuracy (*p*s ≥ .335) nor did perspective moderate the main effects of stakes or scarcity on either dependent variable (*p*s ≥ .173).

Putting the three measures of preferences into relation, we observed a stronger association between the separate accuracy measure and the trade-off measure ($|r_s(4{,}979)| = .65$, $p < .001$, |95% CI| [0.67, 0.63]) compared to a weaker association between the separate interpretability measure and trade-off measure, $r_s(4{,}978) = .57$, $p < .001$, 95% CI [0.55, 0.59]. This might indicate spillover effects of different strengths from the trade-off to the subsequent separate measures, whereby participants' evaluations on the separate measures were influenced by their trade-off preferences and more so for accuracy compared to interpretability. Further support for this interpretation comes from a negative correlation between the two separate measures ($r_s(4{,}979) = -.35$, $p < .001$, 95% CI [-0.37, -0.32]), which implies that stronger valuations of separately measured accuracy came with lower valuations of separately measured interpretability. Finally, we observed significant order-effects on the trade-off ($\chi^2(1) = 12.06$, $p < .001$) and separate interpretability measures ($\chi^2(1) = 5.13$, $p = .024$), but not on the separate accuracy measure ($\chi^2(1) = 0.25$, $p = .614$) when we added a dummy-predictor to the respective mixed effects models (main effects for *stakes* and *scarcity* remained significant). These were driven by a stronger preference for interpretability amongst those participants who encountered this attribute first across instructions and measures.

Pre- & post-task support for ML. Responses did not differ between pre- and post-task measurement ($b = -0.08$, $p = .282$). Summarizing across times of measurement, participants overall supported the use of AI, averaging at 3.94 which exceeded the scale-midpoint, $t(497) = 24.76$, $p < .001$, $d = 1.11$ (see *Table SI 19*).

	Dependent variable:
	Support for ML
Post-Task	−0.082
	(0.076)
Constant	3.985***
	(0.054)
Observations	498
Adjusted R^2	0.0003
Note:	*p<0.05; **p<0.01; ***p<0.001

Table SI 19. Pre- and Post-Task Support for ML (Study 4). P-values are determined by a two-sided t-test with no adjustment for multiple comparisons: $p_{Post\text{-}Task} = .282$; $p_{Constant} < .001$. Standard errors are included in parentheses.

Model with general controls. Main effects for *stakes* and *scarcity* were robust to controlling for gender, age, education, income, pre-task support for ML, awareness of AI bias, and computer science knowledge (see *Table SI 20*).

	Dependent variable:
	Trade-Off Preferences
Stakes	−0.513***
	(0.048)
Scarcity	−0.279***
	(0.048)
Stakes x Scarcity	0.028
	(0.068)
Gender	−0.057
	(0.089)
Age	−0.005
	(0.004)
Education	0.062*
	(0.027)
Income	0.001
	(0.019)
Pre-Task Support for ML	−0.060
	(0.050)
Heard about Bias in AI	−0.152
	(0.112)
CS (Some Programming Experience)	0.207
	(0.115)
CS (College Course)	−0.011
	(0.111)
CS (Undergraduate Degree)	−0.056
	(0.151)
CS (Graduate Degree)	0.518**
	(0.159)
Constant	0.353
	(0.333)
Observations	4,959
Log Likelihood	−8,114.154
Akaike Inf. Crit.	16,262.310
Bayesian Inf. Crit.	16,372.960
Note:	*p<0.05; **p<0.01; ***p<0.001

Table SI 20. Linear Mixed Model: Effect of Stakes, Scarcity on Trade-Off Preferences with General Controls (Study 4). P-values are determined by a two-sided t-test (using Satterthwaite's method for denominator degrees of freedom) with no adjustment for multiple comparisons: $p_{Stakes} < .001$; $p_{Scarcity} < .001$; $p_{Stakes\ x\ Scarcity} = .677$; $p_{Gender} = .519$; $p_{Age} = .242$; $p_{Education} = .024$; $p_{Income} = .951$; $p_{Pre\text{-}Task\ Support\ for\ ML} = .226$; $p_{Heard\ about\ Bias\ in\ AI} = .176$; $p_{CS\ (Some)} = .071$; $p_{CS\ (College)} = .923$; $p_{CS\ (Undergrad)} = .712$; $p_{CS\ (Grad)} = .002$; $p_{Constant} = .290$. Standard errors are included in parentheses.

Model with explanatory controls. When we added explanatory variables to our mixed effects model predicting preferences over accuracy versus interpretability, main effects for stakes and scarcity remained significant. We observed a significant main effect for *reversibility,* whereby demand for accuracy over explainability was less pronounced in applications for which they considered the AI's decisions to be reversible. There was also a significant main effect for *human expertise,* whereby demand for accuracy over explainability was more pronounced in applications they considered to require a high level of human expertise (see *Table SI 21*).

	Dependent variable:
	Trade-Off Preferences
Stakes	−0.516***
	(0.047)
Scarcity	−0.280***
	(0.047)
Stakes x Scarcity	0.034
	(0.067)
Reversibility	0.079***
	(0.017)
Human Expertise	−0.102***
	(0.022)
Personal Affectedness	0.032
	(0.018)
No. People Affected	−0.012
	(0.011)
Constant	0.123
	(0.138)
Observations	4,959
Log Likelihood	−8,095.685
Akaike Inf. Crit.	16,213.370
Bayesian Inf. Crit.	16,284.970
Note:	*p<0.05; **p<0.01; ***p<0.001

Table SI 21. Linear Mixed Model: Effect of Stakes, Scarcity on Trade-off Preferences with Explanatory Controls (Study 4). P-values are determined by a two-sided t-test (using Satterthwaite's method for denominator degrees of freedom) with no adjustment for multiple comparisons: $p_{Stakes} < .001$; $p_{Scarcity} < .001$; $p_{Stakes\ x\ Scarcity} = .614$; $p_{Reversibility} < .001$; $p_{Human\ Expertise} < .001$; $p_{Personal\ Affectedness} = .078$; $p_{No.\ People\ Affected} = .247$; $p_{Constant} = .373$. Standard errors are included in parentheses.

Supplementary Notes

Materials

General note: emphasis (e.g., bolding or use of italics) in the instructions is reported here in the same format as it was presented to participants.

Study 1A

Participants read brief descriptions of AI applications (see below). For each one, they submitted their answers on the following dependent variable:

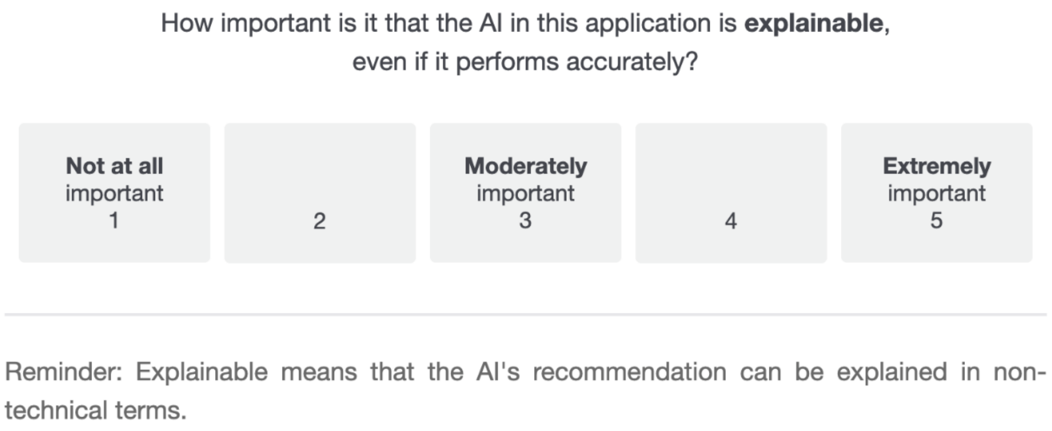

Participants were randomly allocated to either the *recommend* or *decide* condition. Correspondingly, each participant saw only one version of the vignettes (see *Table SI 22*). Note that the 'citizen surveillance' and 'virtual assistants' applications were only presented as *decide* versions, because the *recommend* versions did not seem to make sense.

We also collected additional variables. Participants answered the question "How much do you oppose or support the use of AI" both before and after completing the main task on a 5-point categorical scale ranging from "strongly oppose" to "strongly support". After providing their ratings for the applications, participants indicated on a 5-point categorical scale how important they considered the respective motives, ranging from "not at all important" to "extremely important":

Explain to justify: "We need explainable AI to ensure that its decisions were made fairly and ethically."
Explain to verify: "We need explainable AI to ensure that its decisions are free of errors and were made accurately."
Explain to improve: "We need explainable AI to be able to improve it further."
Explain to discover: "We need explainable AI to learn new facts and gather new information, which ultimately advances knowledge."

We asked how likely they considered it that their main occupation would be replaced by AI at some point on a 5-point categorical scale ranging from "extremely unlikely" to "extremely likely." Also, we asked participants their knowledge of computer science (i.e., graduate degree, undergraduate degree, taken a college-level course, some programming experience, or none of the educational or work experiences described prior). Finally, we collected demographic information for gender, age, education, and income.

Table SI 22. *Recommend* and *Decide* Versions across Different AI Applications.

AI Application	Recommend Version	Decide Version
Adaptive Education[5,6]	AI recommends to a school teacher what learning goals to set for a given student.	AI sets on behalf of a school teacher the learning goals for a given student.
Distribution of Medical Treatment[7,8]	AI recommends to a healthcare provider which patients should be given expensive and/or scarce medical treatments.	AI selects on behalf of a healthcare provider the patients who will be given expensive and/or scarce medical treatments.
Grading Essays[9,10]	AI recommends to a teacher what grade to assign for a student's essay.	AI assigns a grade for a student's essay on behalf of a teacher.
Cyber Security[11]	AI recommends to a computer user how to respond to a hacking attempt.	AI responds on behalf of a computer user to a hacking attempt.
Citizen Surveillance[12,13]		AI surveils suspected criminal offenders through CCTV on behalf of law enforcement authorities.
Organizing Pictures[14,15]	AI recommends to a photo assistant user how to organize their pictures -- e.g., by faces depicted.	AI organizes pictures on behalf of a photo assistant user -- e.g., by faces depicted.
Financial Fraud Detection[16,17]	AI recommends to fiscal authorities what firms might be evading taxes.	AI imposes audits on behalf of fiscal authorities on firms that might be evading taxes.
Processing of Financial Loans[18,19]	AI recommends to a bank whether to approve or deny applications for financial loans or credit.	AI approves or denies applications for financial loans or credit on behalf of a bank.
Gaming[20,21]	AI recommends optimized moves to human game-players.	AI executes optimized moves on behalf of human game-players.
Job Hiring[22–24]	AI recommends to an employer the best candidate to hire for a job vacancy.	AI hires on behalf of an employer the best candidate for a job vacancy.
Historic Photograph Restoration[25]	AI recommends to a photo lab technician how to repair historic photographs that are damaged.	AI repairs on behalf of a photo lab technician historic photographs that are damaged.
Improving Vegetables' Flavor[26]	AI recommends to a greenhouse operator how to optimize the environment of vegetable plants to improve their flavor.	AI optimizes on behalf of a greenhouse operator the environment of vegetable plants to improve their flavor.
Insurance Pricing[27]	AI recommends to an insurance company what insurance rate to set for a customer.	AI sets on behalf of an insurance company the insurance rate for a customer.
Legal Case Research[28,29]	AI recommends to a lawyer which legal information -- e.g., court decisions or existing laws -- is relevant for a given legal case.	AI determines on behalf of a lawyer which legal information -- e.g., court decisions or existing laws -- is relevant for a given legal case.
Facebook Content Moderation[30–32]	AI recommends to a Facebook employee what posts -- e.g., those containing nudity or terrorist propaganda -- to take down from the platform	AI takes down social media posts -- e.g., those containing nudity or terrorist propaganda -- on behalf of a Facebook employee.
Medical Diagnosing[33–35]	AI recommends to a doctor what disease a patient might be suffering from.	AI establishes on behalf of a doctor what disease a patient might be suffering from.

Operation of Military Weapons[36,37]	AI recommends to a military agent which targets to place under fire.	AI decides on behalf of a military agent which targets to place under fire.
Online Shopping[38]	AI recommends products to a customer that they might need or enjoy.	AI buys products on behalf of a customer that they might need or enjoy.
Operating Private Cars[39]	AI recommends to a human driver how to operate their car -- e.g., how to back their car into a parking spot.	AI operates a car on behalf of a human driver -- e.g., it backs their car into a parking spot.
Parole Review[40–42]	AI recommends to a judicial officer whether or not to release an inmate from prison.	AI decides on behalf of a judicial officer whether or not to release an inmate from prison.
Political News Reporting[43,44]	AI recommends to a journalist how data from a political event can be turned into a news report.	AI creates a news report on behalf of a journalist using data from a political event.
Romantic Partner Search[45]	AI recommends compatible romantic partners to a dating app user.	AI selects compatible romantic partners on behalf of a dating app user.
Scientific Discoveries[46]	AI recommends to scientists what experiments might lead to new discoveries -- e.g., the discovery of new materials.	AI runs on behalf of scientists experiments that might lead to new discoveries -- e.g., the discovery of new materials.
Speech Recognition-based Virtual Assistants[47,48]		AI interacts with a person through speech-recognition-based virtual assistants such as Amazon's Alexa, Apple's Siri, or Google Home -- e.g., by turning on the music when the person tells them to do so.
Sports News Reporting[49,50]	AI recommends to a journalist how data from a sports event can be turned into a news report.	AI creates a news report on behalf of a journalist using data from a sports event.
Processing of Unemployment Benefits[51,52]	AI recommends to a civil servant what level of government assistance an unemployed person is eligible to receive.	AI determines on behalf of a civil servant what level of government assistance an unemployed person is eligible to receive.
Immigration Application Processing[53,54]	WAI recommends to a civil servant whether an immigration application should be approved or denied.	AI approves or denies an immigration application on behalf of a civil servant.

Studies 1B and 1C

For Studies 1B and 1C, we used the same vignettes as in Study 1A, but only the *decide* versions. Furthermore, we added two additional vignettes with applications that featured in Studies 2-4:

Allocating standby flight passengers
AI decides which standby passengers are allowed to board an international flight departing in 30 minutes.

Prioritizing first responders for a hurricane
AI decides which locations within a U.S. county will be prioritized to receive aid from first responders --e.g., police officers and paramedics -- when a hurricane strikes.

Different to Study 1A, participants reported their attitudes towards AI interpretability on a continuous slider measure instead of a discrete scale:

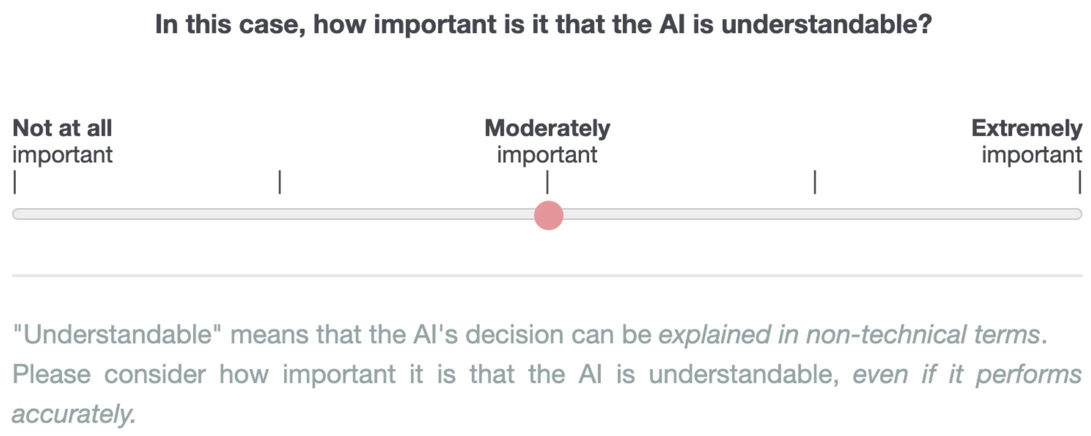

Study 2

Participants went through five different AI applications described in short vignettes. We manipulated stakes and scarcity independently for each vignette, resulting four versions (see below), for each of which participants indicated their attitudes towards interpretability on the following slider scale:

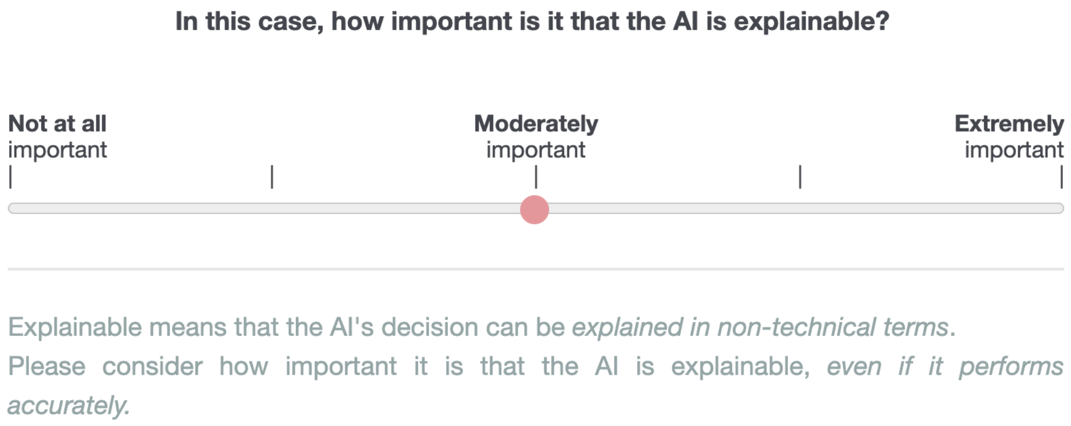

Vaccination allocation

It is flu season. An AI decides whether or not a citizen will get a vaccine.

	Low Stakes	High Stakes
Low Scarcity	In this case, **the vaccine supply is abundant**. Because the vaccine is very cheap, easy to produce, and can be stored at any temperature, **the vaccine can be stocked in large quantities.**<br><br>This particular **vaccine protects against a mild variant of the flu**.	In this case, **the vaccine supply is abundant**. Because the vaccine is very cheap, easy to produce, and can be stored at any temperature, **the vaccine can be stocked in large quantities**.<br><br>This particular **vaccine protects against a deadly variant of the flu**.
High Scarcity	In this case, **the vaccine supply is very limited**. Because the vaccine is very expensive, laborious to produce, and must be stored at low temperatures, **the vaccine cannot be stocked in large quantities**.<br><br>This particular **vaccine protects against a mild variant of the flu**.	In this case, **the vaccine supply is very limited**. Because the vaccine is very expensive, laborious to produce, and must be stored at low temperatures, **the vaccine cannot be stocked in large quantities**.<br><br>This particular **vaccine protects against a deadly variant of the flu.**

Allocating standby flight passengers

An international flight is departing in 30 minutes. An AI decides which standby passengers are allowed to board. Note: Standby passengers are passengers who do have a ticket for a flight to a particular destination, but they do not have a reservation for a specific flight.

	Low Stakes	High Stakes
Low Scarcity	In this case, **there are 10 standby passengers, and 9 seats are available.**<br><br>The **next flight to the same destination leaves one hour later** and it has 20 available seats**.**	In this case, **there are 10 standby passengers, and 9 seats are available.**<br><br>The **next flight to the same destination does not leave until the following day** and it has 20 available seats**.**
High Scarcity	In this case, **there are 10 standby passengers, but only one seat is available.**<br><br>The **next flight to the same destination leaves one hour later** and it has 20 available seats**.**	In this case, **there are 10 standby passengers, but only one seat is available.**<br><br>The **next flight to the same destination does not leave until the following day** and it has 20 available seats**.**

Prioritizing first responders for a hurricane

A U.S. county (population: 30,000) is preparing for a hurricane. An AI decides which locations within the county will be prioritized to receive aid from first responders, such as police officers, and paramedics, when the hurricane strikes.

	Low Stakes	High Stakes
Low Scarcity	In this case, **the county's first responder services are more than adequately staffed.** Therefore, **first responders will be able to provide help to most locations** within the county.<br><br>The **hurricane is expected to be minor, causing minimal damage** across a number of locations.	In this case, **the county's first responder services are more than adequately staffed.** Therefore, **first responders will be able to provide help to most locations** within the county.<br><br>The **hurricane is expected to be severe, causing catastrophic damage** across a number of locations.
High Scarcity	In this case, **the county's first responder services are under-staffed**. Therefore, **first responders will only be able to provide help to very few locations** within the county.<br><br>The **hurricane is expected to be minor, causing minimal damage** across a number of locations.	In this case, **the county's first responder services are under-staffed**. Therefore, **first responders will only be able to provide help to very few locations** within the county.<br><br>The **hurricane is expected to be severe, causing catastrophic damage** across a number of locations.

Reviewing insurance claims

An AI decides whether insurance claims get accepted or rejected.

	Low Stakes	High Stakes
Low Scarcity	In this case, a claim is being filed with an **insurance company that has very lax criteria** for accepting or rejecting a given claim. As a result, **around 90% of insurance claims are approved.**<br><br>The insurance company reviews **claims involving damages between $100 and $200**.	In this case, a claim is being filed with an **insurance company that has very lax criteria** for accepting or rejecting a given claim. As a result, **around 90% of insurance claims are approved.**<br><br>The insurance company reviews **claims involving damages between $40,000 and $50,000**.
High Scarcity	In this case, a claim is being filed with an **insurance company that has very strict criteria** for accepting or rejecting a given claim. As a result, **around 10% of insurance claims are approved.**<br><br>The insurance company reviews **claims involving damages between $100 and $200**.	In this case, a claim is being filed with an **insurance company that has very strict criteria** for accepting or rejecting a given claim. As a result, **around 10% of insurance claims are approved.**<br><br>The insurance company reviews **claims involving damages between $40,000 and $50,000**.

Hiring decisions

An AI makes hiring decisions on behalf of a global company.

	Low Stakes	High Stakes
Low Scarcity	In this case, **the company is thriving and needs to expand, with plans to recruit additional staff** in the near future**.** Hence, **various branches of the company are recruiting for numerous positions**.<br><br>The **positions are honorary**, so a prospective **employee would not expect to earn enough from this job to live on.**	In this case, **the company is thriving and needs to expand, with plans to recruit additional staff** in the near future**.** Hence, **various branches of the company are recruiting for numerous positions.**<br><br>The **positions are salaried**, so a prospective **employee would expect to earn enough from this job to live on**.
High Scarcity	In this case, **the company is thriving and satisfied at its current size, with no plans to recruit additional staff** in the near future. However, because a staff member retired, **a local branch of the company is recruiting for a single position.**<br><br>The **position is honorary,** so a prospective **employee would not expect to earn enough from this job to live on**.	In this case, **the company is thriving and satisfied at its current size, with no plans to recruit additional staff** in the near future. However, because a staff member retired, **a local branch of the company is recruiting for a single position.**<br><br>The **position is salaried,** so a prospective **employee would expect to earn enough from this job to live on**.

We also collected additional variables. Participants answered the question “How much do you oppose or support the use of AI” both before and after completing the main task on a 5-point continuous scale ranging from “strongly oppose” to “strongly support”. Then, participants were asked whether they had heard about “bias” in AI. If so, they were asked whether their ratings about explainability were influenced by what they had heard about bias in AI. We asked how likely they considered it that their main occupation would be replaced by AI at some point on a 5-point continuous scale ranging from “extremely unlikely” to “extremely likely.” Also, we asked participants their knowledge of computer science (i.e., graduate degree, undergraduate degree, taken a college-level course, some programming experience, or none of the educational or work experiences described prior). Finally, we collected demographic information for gender, age, education, income, political interest (on a 7-point categorical scale ranging from “not at all interested in politics” to “very interested in politics”), economic and social political beliefs (on a 7-point categorical scale ranging from “very liberal / left” to “very conservative / right”), and political party.

Study 3A

The extensive introductory instructions for Study 3A are provided in the pre-registration available here on OSF.

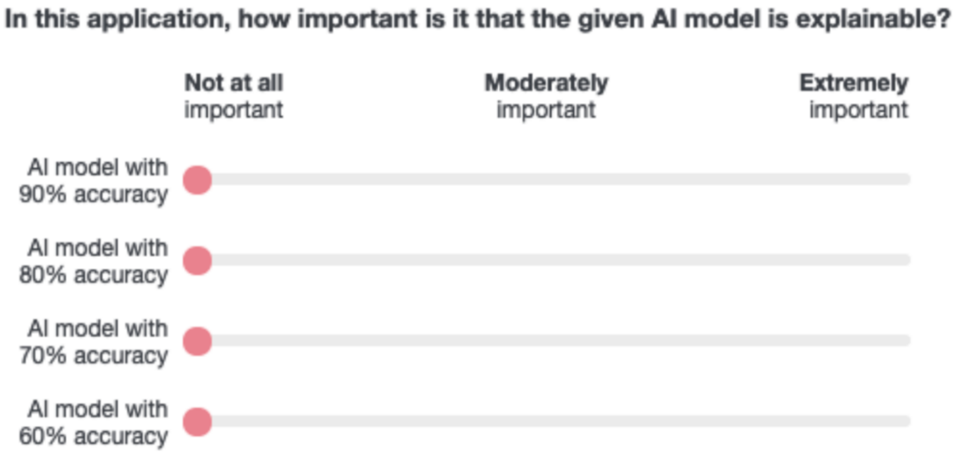

We used only the general versions of the vignettes from Studies 2-4 (see below), each of which were evaluated for four separate AI models that differed in accuracy:

Vaccination allocation
It is flu season. An AI decides whether or not a citizen will get a vaccine.

Allocating standby flight passengers
An international flight is departing in 30 minutes. An AI decides which standby passengers are allowed to board. Note: Standby passengers are passengers who do have a ticket for a flight to a particular destination, but they do not have a reservation for a specific flight.

Prioritizing first responders for a hurricane
A U.S. county (population: 30,000) is preparing for a hurricane. An AI decides which locations within the county will be prioritized to receive aid from first responders, such as police officers, and paramedics, when the hurricane strikes.

Reviewing insurance claims
An AI decides whether insurance claims get accepted or rejected.

Hiring decisions
An AI makes hiring decisions on behalf of a global company.

We also collected additional variables. Participants answered the question "How much do you oppose or support the use of AI" both before and after completing the main task on a 5-point continuous scale ranging from "strongly oppose" to "strongly support". After providing their ratings for the applications, they answered the below questions for general versions of each application:
Human accuracy: "How accurately would a human decision-maker perform?"
Reversibility: "How reversible is a decision performed by the AI?"
Human expertise: "What level of expertise would be required for a human to perform the decision instead of the AI?"
Personal affectedness: "How likely is it that such a decision would affect you personally?"
Number of people affected: "How many people will be affected by the decisions performed by the AI?"

Then, participants were asked whether they had heard about "bias" in AI. We asked how likely they considered it that their main occupation would be replaced by AI at some point on a 5-point continuous scale ranging from "extremely unlikely" to "extremely likely." Also, we asked participants their knowledge of computer science (i.e., graduate degree, undergraduate

degree, taken a college-level course, some programming experience, or none of the educational or work experiences described prior). Finally, we collected demographic information for gender, age, ethnicity, education, income, political interest (on a 7-point categorical scale ranging from “not at all interested in politics” to “very interested in politics”), economic and social political beliefs (on a 7-point categorical scale ranging from “very liberal / left” to “very conservative / right”), and political party.

Study 3B

Participants went through five different AI applications described in short vignettes that were the same as in Study 2. Correspondingly, for each application, there were four different versions according to the *stakes* (low vs. high) and *scarcity* (low vs. high) manipulation and, according to the full within-subject design of Study 3B, each participant saw all four versions of each application. Each version was presented on a separate page. We randomized the order of applications across participants and the order of application-versions both within and across participants.

For each application and version, participants submitted their answers on the following dependent variable (note that the direction of the dependent variable and order of attributes was counterbalanced across participants):

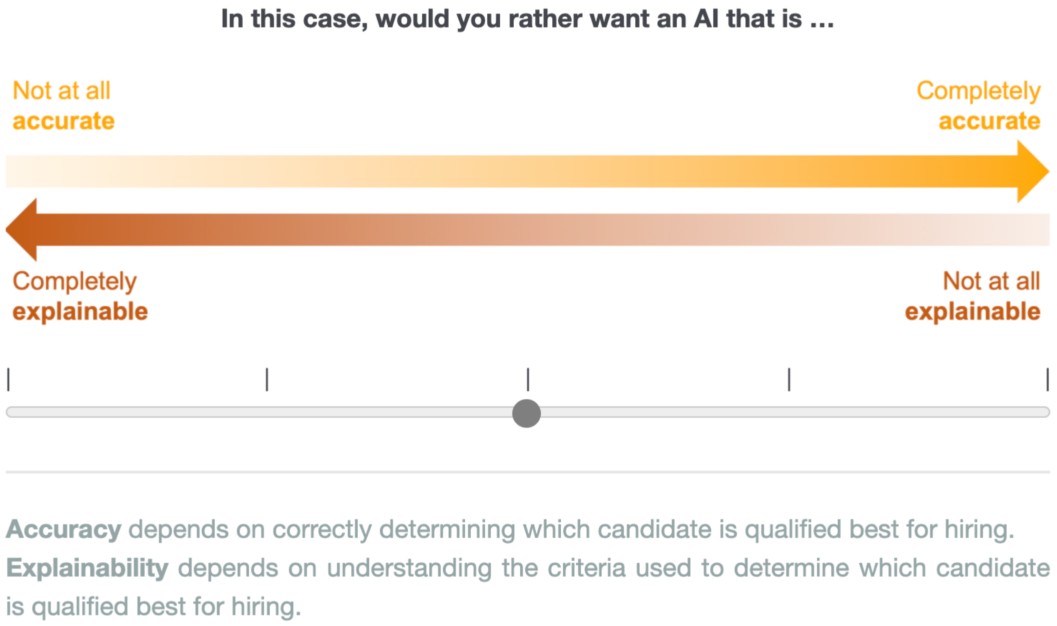

We also collected additional variables. Participants answered the question “How much do you oppose or support the use of AI” both before and after completing the main task on a 5-point continuous scale ranging from “strongly oppose” to “strongly support”. After providing their ratings for the applications, they answered the below questions for general versions of each application:

Reversibility: “How reversible is a decision performed by the AI?”
Human expertise: “What level of expertise would be required for a human to perform the decision instead of the AI?”
Personal affectedness: “How likely is it that such a decision would affect you personally?”
Number of people affected: “How many people will be affected by the decisions performed by the AI?”

Then, participants were asked whether they had heard about “bias” in AI. We asked how likely they considered it that their main occupation would be replaced by AI at some point on a 5-point continuous scale ranging from “extremely unlikely” to “extremely likely.” Also, we asked participants their knowledge of computer science (i.e., graduate degree, undergraduate degree, taken a college-level course, some programming experience, or none of the educational or work experiences described prior). Finally, we collected demographic information for gender, age, education, income, political interest (on a 7-point categorical

scale ranging from “not at all interested in politics” to “very interested in politics”), economic and social political beliefs (on a 7-point categorical scale ranging from “very liberal / left” to “very conservative / right”), and political party.

Study 3C

We used the same dependent variable as in Study 3B. However, according to our between-subjects design, each vignette was presented only once for every participant. To keep the vignettes as consistent as possible across the experimental conditions, the introductory text was the same for all participants (set in black font below), while a given participant would read only one stipulation concerning the stakes and scarcity involved in their case (set in red font below):

Vaccine allocation

It is flu season. An AI decides whether or not a citizen will get a vaccine.

Vaccine supply can be **abundant** or **limited**. Supply is abundant when the vaccine is very cheap, easy to produce, and can be stored at any temperature so that it can be stocked in large quantities. Supply is limited when the vaccine is very expensive, laborious to produce, and must be stored at low temperatures so that it cannot be stocked in large quantities.

Some vaccines protect humans against **mild** variants of the flu, while other vaccines protect humans against **deadly** variants of the flu.

In this case, vaccine supply is abundant. The vaccine protects against a deadly variant of the flu.

In this case, vaccine supply is abundant. The vaccine protects against a mild variant of the flu.

In this case, vaccine supply is limited. The vaccine protects against a deadly variant of the flu.

In this case, vaccine supply is limited. The vaccine protects against a mild variant of the flu.

Allocating standby flight passengers

An international flight is departing in 30 minutes. An AI decides which standby passengers are allowed to board. Note: Standby passengers are passengers who do have a ticket for a flight to a particular destination, but they do not have a reservation for a specific flight.

A flight may have **few** or **many** seats available for standby passengers.
Sometimes, the next flight to the same destination leaves **soon after** (e.g., within an hour), but other times it might only leave the **following day**.

In this case, there are 10 standby passengers, and 9 seats are available. The next

flight to the same destination leaves one hour later and it has 20 available seats.

In this case, there are 10 standby passengers, and 9 seats are available. The next flight to the same destination leaves the following day and it has 20 available seats.

In this case, there are 10 standby passengers, and only one seat is available. The next flight to the same destination leaves one hour later and it has 20 available seats.

In this case, there are 10 standby passengers, and only one seat is available. The next flight to the same destination leaves the following day and it has 20 available seats.

Prioritizing first responders for a hurricane

A U.S. county (population: 30,000) is preparing for a hurricane. An AI decides which locations within the county will be prioritized to receive aid from first responders, such as police officers, and paramedics when the hurricane strikes.

In some counties, first responder services are **more than adequately staffed** and will be able to provide help to most locations within the county. In other counties, first responder services are **understaffed** and will only be able to provide help to very few locations within the county.

Sometimes, a hurricane can be expected to cause **minimal damage**, while other times a hurricane might be expected to cause **catastrophic damage**.

In this case, the county's first responder services are more than adequately staffed and the hurricane is expected to cause minimal damage across a number of locations.

In this case, the county's first responder services are more than adequately staffed and the hurricane is expected to cause catastrophic damage across a number of locations.

In this case, the county's first responder services are understaffed and the hurricane is expected to cause minimal damage across a number of locations.

In this case, the county's first responder services are understaffed and the hurricane is expected to cause catastrophic damage across a number of locations.

Reviewing insurance claims

An AI decides whether insurance claims get accepted or rejected.

Some insurance companies have **very lax criteria** for accepting or rejecting a given claim, resulting in around 90% of insurance claims being approved. Other insurance companies have **very strict criteria** for accepting or rejecting a given claim, resulting in around 10% of insurance claims being approved.

And while some insurance companies review claims involving **smaller damages** between $100 and $200, others review claims involving **larger damages** between $40,000 and $50,000.

In this case, a claim is filed with an insurance company that has very lax criteria, approving around 90% of claims, and that reviews claims involving smaller damages between $100 and $200.

In this case, a claim is filed with an insurance company that has very lax criteria, approving around 90% of claims, and that reviews claims involving larger damages between $40,000 and $50,000.

In this case, a claim is filed with an insurance company that has very strict criteria, approving around 10% of claims, and that reviews claims involving smaller damages between $100 and $200.

In this case, a claim is filed with an insurance company that has very strict criteria, approving around 10% of claims, and that reviews claims involving larger damages between $40,000 and $50,000.

Hiring decisions

An AI makes hiring decisions on behalf of a global company.

Some companies are thriving and need to expand, with plans to recruit additional staff in the near future for **numerous positions**. Other companies are thriving and satisfied at their current size, with no plans to recruit additional staff in the near future so that they may only occasionally recruit for a **single position**.

And while some positions are **honorary**, such that a prospective employee would not expect to earn enough from the job to live on, other positions are **salaried**, such that a prospective employee would expect to earn enough from the job to live on.

In this case, a company is recruiting for numerous positions, which are honorary so that a prospective employee would not expect to earn enough from this job to live on.

In this case, a company is recruiting for numerous positions, which are salaried so that a prospective employee would expect to earn enough from this job to live on.

In this case, a company is recruiting for a single position which is honorary so that a prospective employee would not expect to earn enough from this job to live on.

In this case, a company is recruiting for a single position, which is salaried so that a prospective employee would expect to earn enough from this job to live on.

Study 3D (only in SI)

We used the same dependent variable as in Studies 3A and 3B. The vignettes were the same as in Study 3B, but each participant was presented with only one quadrant of every application.

Study 4 (only in SI)

According to the manipulation of stakeholder perspectives, we modified the vignettes and main dependent variable so that there were *patient* and *agent* versions for each one. Participants also answered two dependent variables for each application and vignette – the first being the tradeoff slider as used in Study 3B, 3B, and 3C; the second asked separately about interpretability and accuracy (the order of the two attributes was counterbalanced for both dependent variables across participants).

[**Question A**, detailed for each application below]

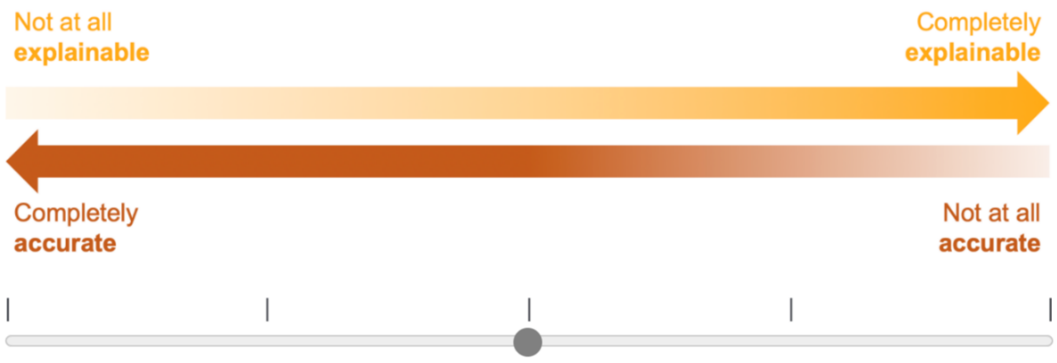

Explainability depends on understanding the criteria used to determine which standby passengers need to board urgently.
Accuracy depends on correctly determining which standby passengers need to board urgently.

Question B: **In this case, how important is it that the AI is ...**

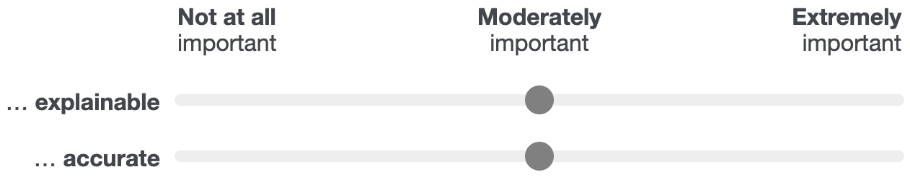

Explainability depends on understanding the criteria used to determine which standby passengers need to board urgently.
Accuracy depends on correctly determining which standby passengers need to board urgently.

<u>Vaccination allocation</u>

It is flu season. An AI decides whether or not a citizen will get a vaccine.

Patient: **You are a citizen seeking to get a vaccine. As a citizen, you are subject to the national healthcare system and thus to the AI making vaccination decisions.**

Agent: **You are the public health director. As public health director, you are responsible for managing the national healthcare system and thus for the AI making vaccination decisions.**

Question A: **As a citizen, would you prefer an AI that is …** *[patient condition]*

Question A: **As a public health director, would you prefer an AI that is …** [*agent condition*]

<u>Allocating standby flight passengers</u>

An international flight is departing in 30 minutes. An AI decides which standby passengers are allowed to board. Note: Standby passengers are passengers who do have a ticket for a flight to a particular destination, but they do not have a reservation for a specific flight.

Patient: **You are a standby passenger who wants to board the flight departing in 30 minutes. As a standby passenger, you are subject to the airline's management of standby passengers and thus to the AI making boarding decisions.**

Agent: **You are the airline director. As airline director, you are responsible for managing standby passengers and thus for the AI making boarding decisions.**

Question A: **As a standby passenger, would you prefer an AI that is …** *[patient condition]*

Question A: **As airline director, would you prefer an AI that is …** [*agent condition*]

<u>Prioritizing first responders for a hurricane</u>

A U.S. county (population: 30,000) is preparing for a hurricane. An AI decides which locations within the county will be prioritized to receive aid from first responders, such as police officers, and paramedics, when the hurricane strikes.

Patient: **You are a local resident hoping to be prioritized for receiving aid from first responders when the hurricane strikes. As a local resident, you are subject to the county-policy for managing first responders and thus to the AI making prioritization decisions.**

Agent: **You are the county's director of emergency services. As director of emergency services, you are responsible for managing first responders when the hurricane strikes and thus for the AI making prioritization decisions.**

Question A: **As a local resident, would you prefer an AI that is …** *[patient condition]*

Question A: **As director of emergency services, would you prefer an AI that is …** [*agent condition*]

__Reviewing insurance claims__

An AI decides whether insurance claims get accepted or rejected.

Patient: **You are an insurance customer who has submitted a claim. As an insurance customer, you are subject to the insurance's management of submitted claims and thus to the AI making acceptance or rejection decisions.**

Agent: **You are the insurance company director. As insurance company director, you are responsible for managing submitted claims and thus for the AI making acceptance or rejection decisions.**

Question A: **As an insurance customer, would you prefer an AI that is …** *[patient condition]*

Question A: **As an insurance director, would you prefer an AI that is …** [*agent condition*]

__Hiring decisions__

An AI makes hiring decisions on behalf of a global company.

Patient: **You are a job candidate seeking to get hired by the company. As a job candidate, you are subject to the company's hiring management and thus to the AI making hiring decisions.**

Agent: **You are the company's recruitment director. As recruitment director, you are responsible for the company's hiring management and thus for the AI making hiring decisions.**

Question A: **As a job candidate, would you prefer an AI that is …** *[patient condition]*

Question A: **As a recruitment director, would you prefer an AI that is …** [*agent condition*]

References

1. Hsee, C. K. The evaluability hypothesis: An explanation for preference reversals between joint and separate evaluations of alternatives. *Organ. Behav. Hum. Decis. Process.* **67**, 247–257 (1996).
2. Hsee, C. K. & Zhang, J. General evaluability theory. *Perspect. Psychol. Sci.* **5**, 343–355 (2010).
3. Biran, O. & Cotton, C. V. Explanation and Justification in Machine Learning : A Survey Or. in (2017).
4. Zhang, B. & Dafoe, A. *Artificial Intelligence: American Attitudes and Trends*. https://papers.ssrn.com/abstract=3312874 (2019).
5. Ayoub, D. Unleashing the power of AI for education. *MIT Technology Review* (2020).
6. Smith, C. S. The Machines Are Learning, and So Are the Students. *The New York Times* (2019).
7. Anukrat Bhansali & Jain, V. Using Machine Learning for Healthcare Resource Allocation in COVID-19: Opportunities and Challenges for LMICs. *Center for Global Development | Ideas to Action* https://www.cgdev.org/blog/using-machine-learning-healthcare-resource-allocation-covid-19-opportunities-and-challenges (2020).
8. Wiggers, K. COVID-19 vaccine distribution algorithms may cement health care inequalities. *VentureBeat* (2020).
9. Smith, T. More States Opting To 'Robo-Grade' Student Essays By Computer. *NPR* (2018).
10. Katz, L. Algorithms are grading student essays across the country. Can this really teach kids how to write better? *Vox* (2019).
11. Janofsky, A. How AI Can Help Stop Cyberattacks. *Wall Street Journal* (2018).
12. Andersen, R. The Panopticon Is Already Here. *The Atlantic* (2020).
13. Avigilon, D. M. S. The New Eyes of Surveillance: Artificial Intelligence and Humanizing Technology. *Wired* (2014).
14. Rhodes, M. Adobe Harnesses AI to Organize Your Photos for You. *Wired* (2016).
15. Biersdorfer, J. D. Organizing Your Unwieldy Photo Collection Is Easier Than You Think. *The New York Times* (2019).
16. Castellanos, S. Visa to Test Advanced AI to Prevent Fraud. *Wall Street Journal* (2019).
17. Nash, K. S. Banks Use AI to Detect if It's Really You. *Wall Street Journal* (2019).
18. Townson, S. AI Can Make Bank Loans More Fair. *Harvard Business Review* (2020).

19. Kahn, J. Can an A.I. algorithm help end unfair lending? This company says yes. *Fortune* (2020).

20. DeepMind co-founder: Gaming inspired AI breakthrough. *BBC News* (2020).

21. Garisto, D. Google AI beats top human players at strategy game StarCraft II. *Nature* (2019) doi:10.1038/d41586-019-03298-6.

22. Heilweil, R. Artificial intelligence will help determine if you get your next job. *Vox* (2019).

23. Polli, F. Using AI to Eliminate Bias from Hiring. *Harvard Business Review* (2019).

24. Harwell, D. A face-scanning algorithm increasingly decides whether you deserve the job. *Washington Post* (2019).

25. AI Restores Photos of '90s Hong Kong Film Stars. *SYNCED* (2019).

26. Knight, W. Machine learning is making pesto even more delicious. *MIT Technology Review* (2019).

27. Murawski, J. Insurers Turn to AI to Better Assess Risk. *Wall Street Journal* (2019).

28. Fitch, A. Would You Trust a Lawyer Bot With Your Legal Needs? *Wall Street Journal* (2020).

29. Toews, R. AI Will Transform The Field Of Law. *Forbes* (2019).

30. Uberti, D. Facebook Turns to Artificial Intelligence for Help During the Pandemic. *Wall Street Journal* (2020).

31. Uberti, D. Why Some Hate Speech Continues to Elude Facebook's AI Machinery. *Wall Street Journal* (2020).

32. Schechner, S. Facebook Boosts AI to Block Terrorist Propaganda. *Wall Street Journal* (2017).

33. Abbott, B. Google AI Beats Doctors at Breast Cancer Detection—Sometimes. *Wall Street Journal* (2020).

34. Parmar, N. AI Holds Promise of Improving Doctors' Diagnoses. *Wall Street Journal* (2017).

35. Rosenbush, S. AI Helps Diagnose Coronavirus. *Wall Street Journal* (2020).

36. Cooke, G. Magic Bullets: The Future of Artificial Intelligence in Weapons Systems. *U.S. Army* https://www.army.mil/article/223026/magic_bullets_the_future_of_artificial_intelligence_in_weapons_systems (2019).

37. Piper, K. Death by algorithm: the age of killer robots is closer than you think. *Vox* (2019).

38. Loten, A. Retailers Use AI to Improve Online Recommendations for Shoppers. *Wall Street Journal* (2020).

39. Kessler, A. Self-Driving Cars Roll Up Slowly. *Wall Street Journal* (2019).

40. Hao, K. AI is sending people to jail—and getting it wrong. *MIT Technology Review* (2019).

41. Tashea, J. Courts Are Using AI to Sentence Criminals. That Must Stop Now. *Wired* (2017).

42. Thompson, D. Should We Be Afraid of AI in the Criminal-Justice System? *The Atlantic* (2019).

43. Peiser, J. The Rise of the Robot Reporter. *The New York Times* (2019).

44. DeGeurin, M. A Startup Media Site Says AI Can Take Bias Out of News. *Vice* (2018).

45. Park, W. How dating app algorithms predict romantic desire. *BBC* (2019).

46. Big pharma is using AI and machine learning in drug discovery and development to save lives. *Insider Intelligence* (2022).

47. Greenwald, T. Digital Assistants Start to Get More Human. *Wall Street Journal* (2018).

48. Merrit, A. Here's what people are really doing with their Alexa and Google Home assistants. *VentureBeat* (2018).

49. Chandler, S. Reuters Uses AI To Prototype First Ever Automated Video Reports. *Forbes* (2020).

50. Beckett, S. Robo-journalism: How a computer describes a sports match. *BBC News* (2015).

51. Hamrin, J. Swedish authorities introduce robots to help social workers. *ComputerWeekly.com* (2019).

52. Martinho-Truswell, E. How AI Could Help the Public Sector. *Harvard Business Review* (2018).

53. Molnar, P. Governments' use of AI in immigration and refugee system needs oversight. *Policy Options* (2018).

54. Akhmetova, R. How AI Is Being Used in Canada's Immigration Decision-Making. *COMPAS* (2020).

55. Malle, B. F. *How the mind explains behavior: Folk explanations, meaning, and social interaction*. (Mit Press, 2006).

56. Lind, E. A. & Tyler, T. R. *The social psychology of procedural justice*. (Springer Science & Business Media, 1988).
