# Supplementary material for: Public attitudes value interpretability but prioritize accuracy in Artificial Intelligence
Source: Nat Commun. 2022 Oct 3;13:5821. doi: 10.1038/s41467-022-33417-3 (PMC9528860; doi:10.1038/s41467-022-33417-3)
Supplement: Supplementary file 3 — Reporting Summary [file 41467_2022_33417_MOESM3_ESM.pdf]

nature portfolio

Corresponding author(s): Anne-Marie Nussberger
Molly Crockett

Last updated by author(s): Aug 22, 2022

Reporting Summary

Nature Portfolio wishes to improve the reproducibility of the work that we publish. This form provides structure for consistency and transparency in reporting. For further information on Nature Portfolio policies, see our Editorial Policies and the Editorial Policy Checklist.

Statistics

For all statistical analyses, confirm that the following items are present in the figure legend, table legend, main text, or Methods section.

n/a	Confirmed	
☐	☒	The exact sample size (*n*) for each experimental group/condition, given as a discrete number and unit of measurement
☐	☒	A statement on whether measurements were taken from distinct samples or whether the same sample was measured repeatedly
☐	☒	The statistical test(s) used AND whether they are one- or two-sided<br>*Only common tests should be described solely by name; describe more complex techniques in the Methods section.*
☐	☒	A description of all covariates tested
☐	☒	A description of any assumptions or corrections, such as tests of normality and adjustment for multiple comparisons
☐	☒	A full description of the statistical parameters including central tendency (e.g. means) or other basic estimates (e.g. regression coefficient) AND variation (e.g. standard deviation) or associated estimates of uncertainty (e.g. confidence intervals)
☐	☒	For null hypothesis testing, the test statistic (e.g. *F*, *t*, *r*) with confidence intervals, effect sizes, degrees of freedom and *P* value noted<br>*Give P values as exact values whenever suitable.*
☒	☐	For Bayesian analysis, information on the choice of priors and Markov chain Monte Carlo settings
☐	☒	For hierarchical and complex designs, identification of the appropriate level for tests and full reporting of outcomes
☐	☒	Estimates of effect sizes (e.g. Cohen's *d*, Pearson's *r*), indicating how they were calculated

Our web collection on statistics for biologists contains articles on many of the points above.

Software and code

Policy information about availability of computer code

Data collection	All studies were implemented using the Qualtrics software's (versions from April 2019 until May 2021) building blocks. Using links that redirected participants to the Qualtrics survey, all data was then collected via Amazon's Mechanical Turk (Studies 1A, 2, 3B, 3D, 4) or Academic Prolific (Studies 1B, 1B, 3A, 3C).
Data analysis	All data analysis was conducted in R and the corresponding code is available at the Open Science Framework under https://doi.org/10.17605/OSF.IO/DQ4VC

For manuscripts utilizing custom algorithms or software that are central to the research but not yet described in published literature, software must be made available to editors and reviewers. We strongly encourage code deposition in a community repository (e.g. GitHub). See the Nature Portfolio guidelines for submitting code & software for further information.

Data

Policy information about availability of data

All manuscripts must include a data availability statement. This statement should provide the following information, where applicable:

- Accession codes, unique identifiers, or web links for publicly available datasets
- A description of any restrictions on data availability
- For clinical datasets or third party data, please ensure that the statement adheres to our policy

All original (de-identified) data is available at the Open Science Framework under https://doi.org/10.17605/OSF.IO/DQ4VC

Field-specific reporting

Please select the one below that is the best fit for your research. If you are not sure, read the appropriate sections before making your selection.

☐ Life sciences ☒ Behavioural & social sciences ☐ Ecological, evolutionary & environmental sciences

For a reference copy of the document with all sections, see nature.com/documents/nr-reporting-summary-flat.pdf

Behavioural & social sciences study design

All studies must disclose on these points even when the disclosure is negative.

Study description

Our manuscript and SI present a total of nine quantitative studies, including a mix of survey (Studies 1B-C) and experimental designs (Studies 1A, 2-4).

Study 1A surveyed attitudes towards AI interpretability using a mixed design, where each participant reported how important they valued AI interpretability for 25 AI applications. Participants were randomly allocated to one of two experimental groups depending on whether they were presented with an application-description where an AI system makes decisions on behalf of a human decision-maker (decide), or an application-description where an AI system makes recommendations to a human decision-maker (recommend).

Studies 1B and 1C surveyed attitudes towards AI interpretability using a within-subjects design, where each participant reported how important they valued AI interpretability for 30 AI applications. There was no experimental treatment.

Study 2 surveyed attitudes towards AI interpretability using a within-subjects design, where each participant reported how important they valued AI interpretability across five AI applications (allocating vaccines, prioritizing first responders, reviewing insurance claims, making hiring decisions, allocating standby passengers). For each of the five AI applications, there were four versions according to a 2 (stakes: low versus high) by 2 (scarcity: yes versus no) within-subjects manipulation.

Study 3A surveyed attitudes towards AI interpretability using a within-subjects design, where each participant reported how important they valued AI interpretability for AI models varying in their perfomances' accuracy (60%, 70%, 80%, 90%). Again, participants were presented with five AI applications (allocating vaccines, prioritizing first responders, reviewing insurance claims, making hiring decisions, allocating standby passengers).

Study 3B surveyed attitudes towards AI interpretability when it trades off with AI accuracy using a within-subjects design, where each participant indicated their attitudes on a slider measure where one end represented a perfectly interpretably but inaccurate AI, and the other slider-end represented the other extreme. Again, participants were presented with five AI applications (allocating vaccines, prioritizing first responders, reviewing insurance claims, making hiring decisions, allocating standby passengers) and with four versions of each application according to a 2 (stakes: low versus high) by 2 (scarcity: low versus high) within-subjects manipulation.

Study 3C also observed attitudes towards AI interpretability when it trades off with AI accuracy, however this time using a between-subjects manipulation of stakes (low versus high) and scarcity low versus high). Again, participants went through five AI applications (allocating vaccines, prioritizing first responders, reviewing insurance claims, making hiring decisions, allocating standby passengers) but, according to the between-subjects manipulation, they saw only one version of it.

Study 3D (SI) was analogous to Study 3C, with the only difference that the instructions did not mention the possible range of stakes or scarcity to participants.

Study 4 explored attitudes towards interpretability and accuracy independently as well as in tandem, and across different stakeholder perspectives. Using a mixed design, participants were presented with AI application descriptions from the perspective of a responsible agent, who oversees the decision, or the perspective of a patient, who is affected by the decision. Participants first indicated their attitudes towards interpretability at the expense of accuracy (as in Studies 3B-3C) and subsequently indicated their attitudes towards interpretability and accuracy separately. Again, participants went through five AI applications (allocating vaccines, prioritizing first responders, reviewing insurance claims, making hiring decisions, allocating standby passengers).

Research sample

Study 1A: We recruited 200 US participants via MTurk, relying on a non-representative convenience sample as this was an initial exploration of non-expert attitudes towards AI interpretability. One duplicate response and 29 participants who failed a comprehension check on their second attempt were excluded from our analyses, leaving a final sample of N = 170. The final sample included 107 males, 60 females, and 3 “other” with an average age of 36.94 (SD = 11.83, SE = 0.91). 20 of the participants’ highest education was high school; 40, some college; 16, a two-year degree; 75, a four-year degree; and 19, a postgrad or another professional degree. The mean income bracket was between $35,001 and $50,000. Most participants (74) had no formal education in computer science; 35 had some programming experience; 45 took a college-level course; 13 held an undergraduate degree; and 3 held a graduate degree in computer science.

Study 1B: We recruited 293 US participants via Academic Prolific. Because we sought to test whether the results from Study 1A generalise across different populations and representative samples of such, we used Prolific’s feature for collecting a representative sample that matches US census data in terms of age by sex by ethnic group proportions in Study 1B. Of those, in line with the pre-registration, we excluded 33 participants who failed comprehension checks on both attempts, or who failed all attention checks. The final sample of N = 258 included 125 males, 131 females, 1 nonbinary person, and 1 who chose “prefer not to say” with an average age of 45.53 (SD = 16.41, SE = 1.02). 2 of the participants’ highest education was less than high school; 22, high school; 49, some college; 17, a two-year degree; 112, a four-year degree; and 56, a postgrad or another professional degree. The mean income

bracket was between $35,001 and $50,000. Most participants (129) had no formal education in computer science; 42 had some programming experience; 65 took a college-level course; 15 held an undergraduate degree; and 7 held a graduate degree in computer science.

Study 1C: Following the same rationale as for Study 1B, we recruited 298 UK participants via Academic Prolific, using the platform's feature for collecting representative samples that match census data in terms of age by sex by ethnic group proportions. Of those, in line with the pre-registration, we excluded 52 participants who failed comprehension checks on both attempts, or who failed all attention checks. The resulting final sample of N = 246 included 118 males and 128 females with an average age of 45.57 (SD = 15.94, SE = 1.02). 28 of the participants' highest education was less than high school; 57, high school; 102, some college; and 59, a two-year degree. The mean income bracket was between $15,001 and $25,000. Most participants (162) had no formal education in computer science; 50 had some programming experience; 18 took a college-level course; 9 held an undergraduate degree; and 7 held a graduate degree in computer science.

Study 2: We recruited 120 US participants via MTurk using a non-representative convenience sample as this study was again exploratory. 36 participants failed comprehension checks on two attempts and were excluded from all analyses, resulting in a final sample of N = 84. The final sample included 46 males, 36 females, 1 "other", and 1 "prefer not to say" with an average age of 37.38 (SD = 11.76, SE = 1.28). 13 of the participants' highest education was high school; 25, some college; 7, a two-year degree; 31, a four-year degree; and 8, a postgrad or another professional degree. The mean income bracket was between $25,001 and $35,000. 5 of the participants had no formal education in computer science; 42 had some programming experience; 14 took a college-level course; 18 held an undergraduate degree; and 5 held a graduate degree in computer science.

Study 3A: We recruited 302 US participants via Academic Prolific, using the platform's feature for collecting representative samples that match census data in terms of age by sex by ethnic group proportions. We collected this data during the revision process at a time when we wanted to ensure comparibility in terms of samples between additionally collected data (at the time Studies 1B, 1C, 3A, 3C). In line with our pre-registration, we excluded 41 participants who failed a comprehension check on two attempts, leaving a final sample of N = 261. The final sample included 124 males, 130 females, 4 nonbinary, and 1 "prefer not to say" with an average age of 44.88 (SD = 15.80, SE = 0.98). 24 of the participants' highest education was high school; 42 some college; 30, a two-year degree; 109, a four-year degree; and 56, a postgrad or another professional degree. The mean income bracket was between $35,001 and $50,000. Most participants (134) had no formal education in computer science; 47 had some programming experience; 60 took a college-level course; 15 held an undergraduate degree; and 5 held a graduate degree in computer science.

Study 3B: We recruited 117 US participants via MTurk using a non-representative convenience sample as this study served as an initial exploration of attitudes towards interpretability-accuracy tradeoffs. To reduce the dropout from failed comprehension checks that we had witnessed in Study 2, we only let those participants proceed to the full survey, who successfully passed comprehension checks. Participants who failed to do so on two attempts were paid a compensation fee for their time until that point and were replaced. After excluding five duplicate responses, this left us with a final sample of N = 112. The final sample included 77 males, 34 females, and 1 "prefer not to say" with an average age of 36.45 (SD = 10.30, SE = 0.97). 14 of the participants' highest education was high school; 22, some college; 11, a two-year degree; 50, a four-year degree; and 15, a postgrad or another professional degree. The mean income bracket was between $35,001 and $50,000. Most participants (45) had no formal education in computer science; 21 had some programming experience; 29 took a college-level course; 7 held an undergraduate degree; and 10 held a graduate degree in computer science.

Study 3C: We recruited 1,501 US participants via Academic Prolific, using the platform's feature for collecting representative samples that match census data in terms of age by sex by ethnic group proportions. Because this study was a replication of Study 3B, we aimed to collect a population-typical sample that would additionally be comparable to the sample recruited in Study 3A. In line with our pre-registration, we excluded 27 incomplete and duplicate responses and 127 participants who failed a comprehension check on two attempts, resulting in a final sample of N = 1,344. The final sample included 720 males, 605 females, 1 nonbinary, and 16 "prefer not to say" with an average age of 39.26 (SD = 14.90, SE = 0.41). 8 of the participants' highest education was less than high school; 103, high school; 279, some college; 106, a two-year degree; 422, a four-year degree; and 423, a postgrad or another professional degree. The mean income bracket was between $35,001 and $50,000. Most participants (610) had no formal education in computer science; 211 had some programming experience; 367 took a college-level course; 44 held an undergraduate degree; and 111 held a graduate degree in computer science.

Study 3D (only in SI): We recruited 1,614 US participants via Academic Prolific, using the platform's feature for collecting representative samples that match census data in terms of age by sex by ethnic group proportions. Again, using a representative sample allowed us to relate more directly to other our findings, in particular Study 3C, and was additionally motivated by this study being a replication of Studies 3B and 3C. In line with our pre-registration, we excluded 22 duplicate cases, 87 participants who did not finish the survey (progress ≤ 85%), and 87 participants who failed a simple comprehension check on two attempts, leaving a final sample of N = 1,418. The final sample included 687 males, 713 females, 13 nonbinary, and 5 "prefer not to say" with an average age of 44.61 (SD = 16.38, SE = 0.44). 5 of the participants' highest education was less than high school; 135, high school; 334, some college; 136, a two-year degree; 499, a four-year degree; 309, a postgrad or another professional degree. The mean income bracket was between $35,001 and $50,000. Most participants (691) had no formal education in computer science; 261 had some programming experience; 337 took a college-level course; 76 held an undergraduate degree; and 53 held a graduate degree in computer science.

Study 4 (only in SI): We recruited 249 participants via MTurk using a non-representative convenience sample as this study was explorative. The final sample included 163 males, 85 females, and 1 "other" with an average age of 35.57 (SD = 9.62, SE = 0.61). 2 of the participants' highest education was less than high school; 28, high school; 50, some college; 24, two-year degree; 118, four-year degree; and 27, postgrad or other professional degree. The mean income bracket was between $35,001 and $50,000. 112 of the participants had no computer science knowledge; 47, some experience; 46, a college degree; 22, an undergraduate degree; and 22, a graduate degree.

Sampling strategy

In view of a lack of relevant effect sizes from (not existent) prior work, for our initial studies we estimated required effect sizes based on our research designs that included repeated answers across all nine studies. For Study 1A (random sampling procedure), which included a between-subjects manipulation and 20 repeated answers for each participant, we set the sampling goal at 100

participants per condition. Studies 1B-1C (stratisfied sampling procedure) sought to replicate main findings of Study 1A, while they did not include the between-subejcts manipulation. To this end, we chose to triple the condition-wise sample size from the initial study, recruiting 300 participants for each of the two replication studies. Study 2 (random sampling procedure) used an experimental design with twenty repeated answers for each participant. Based on our experiences from Study 1A, we anticipated that a substantial share of participants would be excluded for failed comprehension checks and thus decided to recruit 120 participants, aiming for a final sample size of 100. For Study 3A stratisfied sampling procedure), which again used a within-subjects design with five repeated answers for each participant, we recruited the minimum sample size of 300 participant that is required to obtain a representative sample. For Study 3B (random sampling procedure), we followed the same sampling rationale as for Study 2. For Study 3C stratisfied sampling procedure), we based our sample size target on a small effect for scarcity (Ω2= .005) observed in Study 3B, with a formal power analysis indicating that a sample of N = 1,564 would be required to achieve a power of .80 using a between-subjects design. Given that Prolific's feature for collecting representative samples is limited to recruiting at N = 1,500, we set this as our sampling goal. The same applied for Study 3D (only in the SI; stratisfied sampling procedure). Study 4 (only in the SI; random sampling procedure) had a similar design as Study 1A and again we were aiming for recruiting 100 participants for each of the two between subjects conditions, though we collected an additional 50 participants in anticipation of exclusions.

Data collection

Data for all studies was collected online, implemented in Qualtrics, and recruited through MTurk or Academic Prolific. Participants were asked to complete the online surveys in a calm environment and on their own.

Timing

(Note that some data was collected within a single day and hence has the same start and stop dates)

Study 1A: data collected 20/04/2019
Studies 1B-1C: data collected 14/01/2021
Study 2: data collected 24/10/2019
Study 3A: data collected 26-28/05/2021
Study 3B: data collected 15-16/11/2019
Study 3C: data collected 25-27/08/2021
Study 3D (SI): data collected 15-21/01/2021
Study 4 (SI): data collected 11-12/12/2019

Data exclusions

Study 1A: Out of 200 recruited participants, we excluded one duplicate response and 29 participants who failed a comprehension check on their second attempt, leaving a final sample of N = 170.

Study 1B: In line with the study's pre-registration, out of 293 recruited participants, we excluded 33 who failed comprehension checks on both attempts, or who failed all attention checks, leaving a final sample of N = 258.

Study 1C: In line with the study's pre-registration, out of 298 recruited participants, we excluded 52 who failed comprehension checks on both attempts, or who failed all attention checks, leaving a final sample of N = 246.

Study 2: Out of 120 recruited participants we excluded 36 who failed comprehension checks on two attempts, resulting in a final sample of N = 84.

Study 3A: In line with the study's pre-registration, out of 302 recruited participants, we excluded 41 who failed comprehension checks on two attempts, leaving a final sample of N = 261.

Study 3B: We recruited 117 participants and excluded 5 duplicate submissions, leaving a final sample of N = 112.

Study 3C: We recruited 1,501 participants and, in line with our pre-registration, excluded 27 incomplete and duplicate responses and 127 participants who failed a comprehension check on two attempts, resulting in a final sample of N = 1,344.

Study 3D (only in SI): We recruited 1,614 participants and, in line with our pre-registration, excluded 22 duplicate cases, 87 participants who did not finish the survey (progress ≤ 85%), and 87 participants who failed a simple comprehension check on two attempts, leaving a final sample of N = 1,418.

Study 4 (only in SI): 440 participants started the survey, out of which 249 passed all comprehension checks immediately after the instructions. We did not collect data for those participants who failed comprehension checks on two attempts and meanwhile included all 249 who had passed them.

Non-participation

For all of our studies, participants were asked to provide informed consent before they were able to proceed to the instructions. We did not record data for people who did not consent at that point and thus cannot specify the number of participants who declined participation.

Randomization

We used a Qualtrics' inbuilt feature for randomising participants into experimental groups (Studies 1A, 3C, 3D, 4). Studies 1B, 1C, 3A, 3B did not involve experimental groups. For all studies, within-participants repetition (AI application) was randomised using Qualtrics' inbuilt feature as well.

Reporting for specific materials, systems and methods

We require information from authors about some types of materials, experimental systems and methods used in many studies. Here, indicate whether each material, system or method listed is relevant to your study. If you are not sure if a list item applies to your research, read the appropriate section before selecting a response.

Materials & experimental systems

n/a	Involved in the study
☒	☐ Antibodies
☒	☐ Eukaryotic cell lines
☒	☐ Palaeontology and archaeology
☒	☐ Animals and other organisms
☐	☒ Human research participants
☒	☐ Clinical data
☒	☐ Dual use research of concern

Methods

n/a	Involved in the study
☒	☐ ChIP-seq
☒	☐ Flow cytometry
☒	☐ MRI-based neuroimaging

Human research participants

Policy information about studies involving human research participants

Population characteristics	See above (research sample)
Recruitment	As outlined above, participants for all studies were recruited online via MTurk or Academic Prolific. These platforms and our study description, which explicitly invited participants to take part in a study on "attitudes towards Artificial Intelligence", could potentially introduce a self-selection bias for participants who are keen and/or familiar with technology and AI in particular. However, we surveyed participants' computer science expertise and their general attitudes towards AI (the latter at the very start and end of our studies) and find no evidence that these control variables affected participants' attitudes towards AI interpretability. Replicating our key results in representative samples was another measure that minimised potential self-selection bias.
Ethics oversight	All studies were approved by the Yale University Human Subjects Committee (approval number: HSC 2000022385) and participants in each study gave their informed consent before proceeding to the instructions.

Note that full information on the approval of the study protocol must also be provided in the manuscript.
